# Assessing Implicit Theories in Sexual Offending Using Indirect Measures: Feasibility, Reliability, and Incremental Validity

**DOI:** 10.1177/10731911241245009

**Published:** 2024-05-07

**Authors:** Mirthe G. C. Noteborn, Jelle J. Sijtsema, Jaap J. A. Denissen, Stefan Bogaerts

**Affiliations:** 1 Tilburg University, The Netherlands; 2 Fivoor Science and Treatment Innovation, The Netherlands; 3 Utrecht University, The Netherlands

**Keywords:** implicit theories of sexual offending, assessment, implicit association test, implicit relational assessment procedure, relational responding task

## Abstract

This study assessed psychometric qualities of indirect measures assessing Implicit Theories (ITs) of sexual offending: Implicit Association Task (IAT), Implicit Relational Assessment Procedure (IRAP), and Relational Responding Task (RRT). For comparison reasons, aggressive behavior was also assessed. In a male sample from the general population (*N* = 109), we assessed each measure’s (a) feasibility (mean latency, error rate, passing criteria), (b) internal consistency, (c) convergent and discriminant validity, and (d) incremental and predictive validity. Results indicated that no indirect measure met all criteria. Although the IAT was reasonably feasible and reliable in measuring aggression, ITs could not be reliably assessed. The RRT was feasible and somewhat reliable in assessing ITs, whereas the IRAP showed limited feasibility, high task complexity, low reliability, and the presence of a method factor. No measure had incremental predictive validity over the use of self-report measures, although we note that the power to detect such associations was limited. As none of the indirect measures performed satisfactorily on the measured criteria, the use of these measures in clinical practice seems currently unwarranted to assess ITs.

Implicit theories (ITs) about sexual offending are defined as offense-related cognitions and interrelated cognitive assumptions (i.e., theories) that sex offenders implicitly have about their victims, themselves, and the world (Polaschek & Ward, 2002; Ward, 2000; Ward & Keenan, 1999). ITs are considered implicit because they are rarely articulated in a formal sense and are not expressed easily. When measured using direct procedures^1^ such as interviews or self-report measures, ITs and their corollaries (e.g., offense-supported cognitions) have emerged as risk factors for sexual, violent, and general recidivism among sex offenders (e.g., Hanson & Morton-Bourgon, 2005; Helmus et al., 2013). Although direct assessments of ITs predict recidivism, they are hindered by methodological limitations, including the inability to assess introspective constructs (e.g., offense-supportive beliefs about victims) and the possibility of bias due to socially desirable responses or self-presentation. The latter is especially problematic because admitting to, for instance, seeing oneself as sexually entitled or viewing children as sexual beings can have serious consequences for forensic patients, such as prolonged treatment (e.g., Kalmus & Beech, 2005; Wilson, 2009).

To address these shortcomings, latency-based indirect measures have been developed. Here, indirectness refers to the way the concept in question is measured—an outcome is measured in another way than self-assessment (cf. de Houwer, 2006). These measures require participants to respond as quickly as possible to stimuli that appear on a computer screen, with response latency as the outcome (i.e., an implicit measure) rather than the chosen response (i.e., an explicit measure). The current study assessed the ability of three indirect measures, namely, the Implicit Association Task (IAT), the Implicit Relational Assessment Procedure (IRAP), and the Relational Responding Task (RRT), to measure ITs in a feasible, reliable, and valid way.

## Types of ITs

Based on previous studies on self-reported cognitive distortions of sex offenders, Polaschek and Ward (2002) stated that sex offenders differ in their endorsement of victim-oriented ITs. When applied to children as targets, ITs depict that children have sexual needs and desires and actively pursue sexual contact (*Children as Sexual Beings*), and/or depict that sexual activity in itself is unlikely to be harmful to children if it does not include force or threat (*Nature of Harm*). When applied to adult women as targets, ITs can be sexual, such as assuming that women always desire sex, even if it is coerced or violent (*Women as Sex Objects*), or non-sexual, such as assuming that women are inherently different from men, and these differences cannot be easily understood by men (*Women are Unknowable*, later revised as *Women are Dangerous* by Polaschek & Gannon, 2004).

In addition to the four victim-orientated ITs, Ward and colleagues identified three general antisocial ITs (Polaschek & Ward, 2002; Ward, 2000; Ward & Keenan, 1999) revolving around the self, others, and the world. First, one particularly narcissistic IT is that one is entitled to do what one wants, due to feeling superior and being more important than others (*Entitlement*). A second IT is that one has no control over life circumstances or behaviors, including sexually abusive behaviors because these behaviors are mainly externally rather than internally controlled (*Uncontrollability*). A third revolves around a malignant perception of the world, described as a dangerous place where others are considered evil and aggressive (*Dangerous World*). All of these more general ITs contain general antisocial components but may also apply to sexual behaviors and situations.

## Classic Indirect Measures

**Figure 1.**
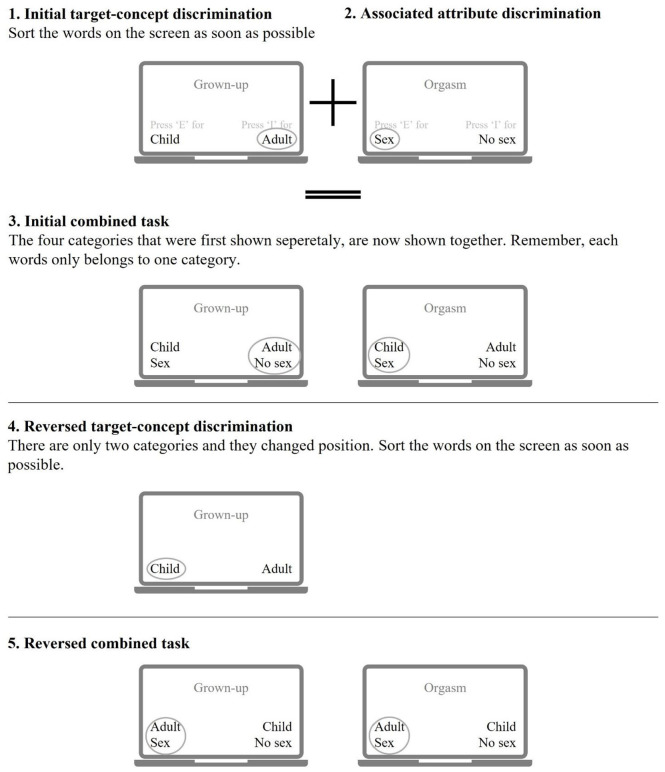
Schematic Picture of the Implicit Association Test (IAT) Procedure.

The Implicit Association Test is one of the most widely used indirect measures (IAT; Greenwald et al., 1998) also in the field of forensic psychology. The IAT assesses the strength of cognitive associations by comparing reaction times to stimuli that contain different combinations of concepts, such as child and sex. Studies have shown that child abusers respond faster than the general population and rapists to the combined categories of child and sex and slower to the combined categories of adult and sex (for a meta-analysis, see Babchishin et al., 2013). For a schematic picture of the IAT (see Figure 1).

Despite the IAT’s frequent use, easy administration (approximately 5 min), and overall acceptable to good internal consistency across fields and domains (e.g., race, politics, self-esteem, violence-related cognitions, attitudes, stereotypes, and sexual attraction to children; Bar-Anan & Nosek, 2014; Blumenthal et al., 2019; Greenwald & Lai, 2020; Schmidt et al., 2015), it is not without criticism. First, the relative strength of the association between two concepts as measured by the IAT does not specify the underlying nature of the measured association (e.g., De Houwer, 2002, 2003). For example, the child–sex association can be representative of the IT Children as Sexual Beings but may also indicate the wish to have sex to conceive a baby (e.g., De Houwer, 2002; Gawronski, 2009). Hence, while some researchers have suggested that the child–sex association is representative of an IT (e.g., Gannon & Polaschek, 2006; Mihailides et al., 2004), others consider the association between child and sex as an indication of sexual interest rather than representing specific beliefs about the child’s sexual desires and needs (e.g., Babchishin et al., 2013; Banse et al., 2010; Kanters et al., 2016).

Moreover, measuring the association between concepts does not allow an evaluation of the value or direction of the relationship linking the concepts (De Houwer et al., 2015). To illustrate, in a study by Mihailides and colleagues (2004) studying IT Entitlement, participants could have responded faster to a combination of “mine” and “sexual” because they like sex, but the statistical association could also indicate a dislike instead of like. Likewise, the association between child and sex is ambiguous, as liking sex with children and disapproving of sex with children are associatively identical (for an in-depth discussion, see Hughes et al., 2011). The representation of a complex belief such as an IT can only be operationalized through a network of several associations, such as between “I,” “entitled,” “sexual contact” (i.e., Sexual Entitlement), or between “sexual contact,” “careful,” “child,” “okay” (i.e., Nature of Harm). However, combining multiple associated constructs is challenging, if not impossible, with IAT-like methods (De Houwer, 2002).

To conclude, despite the measures’ frequent use, ease of administration, and good reliability, associations are ambiguous concerning the qualitative unspecific relation between the concepts involved. Researchers argue that this ambiguity partly explains the weak predictive validity of the IAT (for a meta-analysis on the incremental predictive validity of the IAT across domains such a public policies, interaction with members of certain groups, interpersonal motivations of for instance age, race, sexuality, and religion, see Buttrick et al., 2020).

## Novel Indirect Measures

To avoid interpretational ambiguity and thereby possibly increase predictive value, indirect measures have been developed that require participants to respond *as if* they hold a specific belief. The Implicit Relational Assessment Procedure (IRAP) is an indirect measure focusing on propositional relations (i.e., statements that can be true or false) instead of the strength between associations of concepts (i.e., IAT; Barnes-Holmes et al., 2006). The IRAP is based on the Relational Frame Theory (RFT; see Hayes et al., 2001), which describes how people combine stimuli based on human language and current contextual factors (i.e., relational framing). That is, human language typically not only specifies the strength of a link between stimuli but also uses words or clauses to qualify the type of relationship and the dimension along which they are related. Relating this to the IRAP, it is assumed that when a stimulus is presented, there will be a brief and immediate relational response (e.g., automatic response) based on the relationship between the stimuli and its truth value (i.e., “True” or “False”). The immediate relational response is desired for the IRAP; therefore, the latency of the response is considered the primary outcome. After the desired IRAP outcome, a more extended and elaborated relational response often follows involving cognitive appraisal. This later response can be regarded as the response to, for instance, self-report measures (for more details, see Hughes & Barnes-Holmes, 2013).

**Figure 2.**
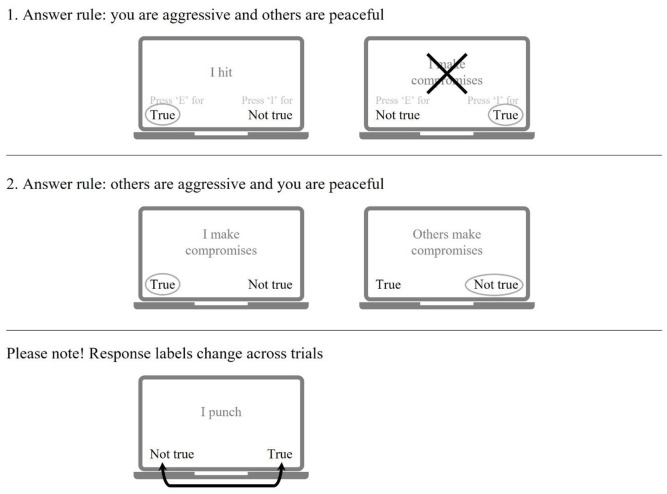
Schematic Picture of the Implicit Relational Assessment Procedure (IRAP).

In a traditional IRAP, participants must respond as if they hold specific beliefs by choosing one of the two response options (True press “X” or False press “M”) when presented with the stimuli. These beliefs are either explicitly stated at the beginning of each trial (e.g., “in this block please respond as if you are sexually entitled”), or participants will be introduced to the specific beliefs by a trial-and-error principle. The stimuli used in the IRAP can be words (e.g., category Child-Adults which will be combined with the Sexy-Not Sexy category using stimuli such as “Sexual,” “Seductive,” etc.; see Dawson et al., 2009) or sentences (e.g., “Children can decide if they want to have sexual contact”; Barnes-Holmes et al., 2006). However, the number of studies using actual sentences is limited. See Figure 2 for a schematic overview of the IRAP procedure. Like the IAT, it is assumed that if participants’ beliefs correspond with the instructed responding belief, responses are faster and with a lower error rate, as a brief and immediate relational response would be correct. If this participant is asked to respond incongruently to his beliefs, his brief and immediate relational responses would result in an error. The “correct” response (in this case: not corresponding to a personally held belief) would result in a more extended and elaborated relational response, increasing his response latency.

To counter the formation of learned response patterns based on the physical location of the response keys (“False” vs. “True”), the physical location of the response options changes on a trial-by-trial basis. Furthermore, to ensure that participants understand and comply with the IRAP instructions and that a brief and immediate relational response is given instead of an extended and elaborated relational response, participants typically must achieve 80% correct responses within an average response time of 2,000 ms. Data from participants who do not meet the speed and accuracy criteria in the practice or test blocks are discarded (for an overview of the procedure see Barnes-Holmes et al., 2006; Hughes & Barnes-Holmes, 2013).

Besides being able to calculate an overall effect (compound *D-score*) indicating whether someone, for instance, feels entitled to have sexual contact (IT Sexual Entitlement), the design of the IRAP makes it possible to calculate four trial-type scores that measure individual traits. For example, the IRAP assessing the IT Sexual Entitlement includes answering affirmative vs. dismissive to statements that one *is* vs. *is not* sexually entitled (e.g., answering affirmative vs. dismissive to statements related to [1] *being sexually entitled*, [2] *not being sexually entitled*, [3] *indicating that women have to obey men’s sexual needs*, and statements that [4] *women can make their own decisions regarding sex*). Due to this design, it is possible to calculate the difference between denying and confirming the above statements in the different conditions, thereby indicating where a possible belief might be.

Dawson and colleagues (2009) used the IRAP to measure the IT Children as Sexual Beings, using the Adult versus Child category labels and target stimuli containing Sexual versus Non-sexual words (Dawson et al., 2009). Both sex offenders and non-offenders showed a preference for adults as sexual over children as sexual (i.e., compound *D-score*). However, when looking at the four individual trial-type scores (Adult—Sexual, Child—Sexual, Adult—Non-Sexual, Child—Non-sexual), sex offenders were less negative toward children as sexual (i.e., responded slightly faster to Child—Sexual trial type) compared with non-offenders.

## Methodological Challenges and Possible New Solutions

One general limitation of using the IRAP is the high dropout rate due to participants failing to meet the criteria of a mean latency of less than 2,000 ms and 80% correct responses across several fields and domains (see Hughes and Barnes-Holmes (2013), Table 1 gives an overview of different studies [e.g., ageism, mood and spider-fear] and their dropout rates). While the high dropout rate can sometimes be avoided by increasing the number of practice trials, lowering the threshold for the percentage of correct responses (e.g., to 65%), or increasing the response latency criteria (e.g., to 3,000-5,000 ms; e.g., Timko et al., 2010; Vahey et al., 2009), this does not always yield favorable results. This is especially apparent in specific (clinical) populations such as forensic samples and individuals with a low educational level (Parling et al., 2012; Vahey et al., 2009). In the study of Dawson et al. (2009), the response latency for the passing criterion was set at 5,000 ms. However, it could be questioned to what extent such latencies can still be considered automatic and uncontrolled, or whether this large time window elicits extended and elaborated relational responses, making it a more direct measure. In addition, low internal consistency is not uncommon for the IRAP (e.g., for a meta-analysis see Hussey & Drake, 2020).

**Table 1:** Passing Criteria for All Indirect Measures of Aggression and Sexual Entitlement.

	Implicit relational assessment procedure		Relational responding task		Implicit association test
	Aggression	Sexual entitlement	Aggression	Sexual entitlement	Aggression
Condition	3.068 ms+80% correct	3.291 ms+80% correct			
*N*	109	109	109	108	109
Participants deleted based on specific passing criteria IRAP	16	25	-	-	-
*n*	93	84	109	108	109
Participants deleted based on 2.5 std. > mean error rate	2	3	3	4	2
Participants deleted based on 10% latencies < 300 ms	0	0	0	0	0
Participants that passed the criteria (n)	91	81	106	104	107
Participants trails latencies > 10.000 ms	4	3	1	10	2

*Note.* One participant did not finish the RRT Entitlement due to time constrains, therefore the total number of participants is 108 instead of 109.

**Figure 3.**
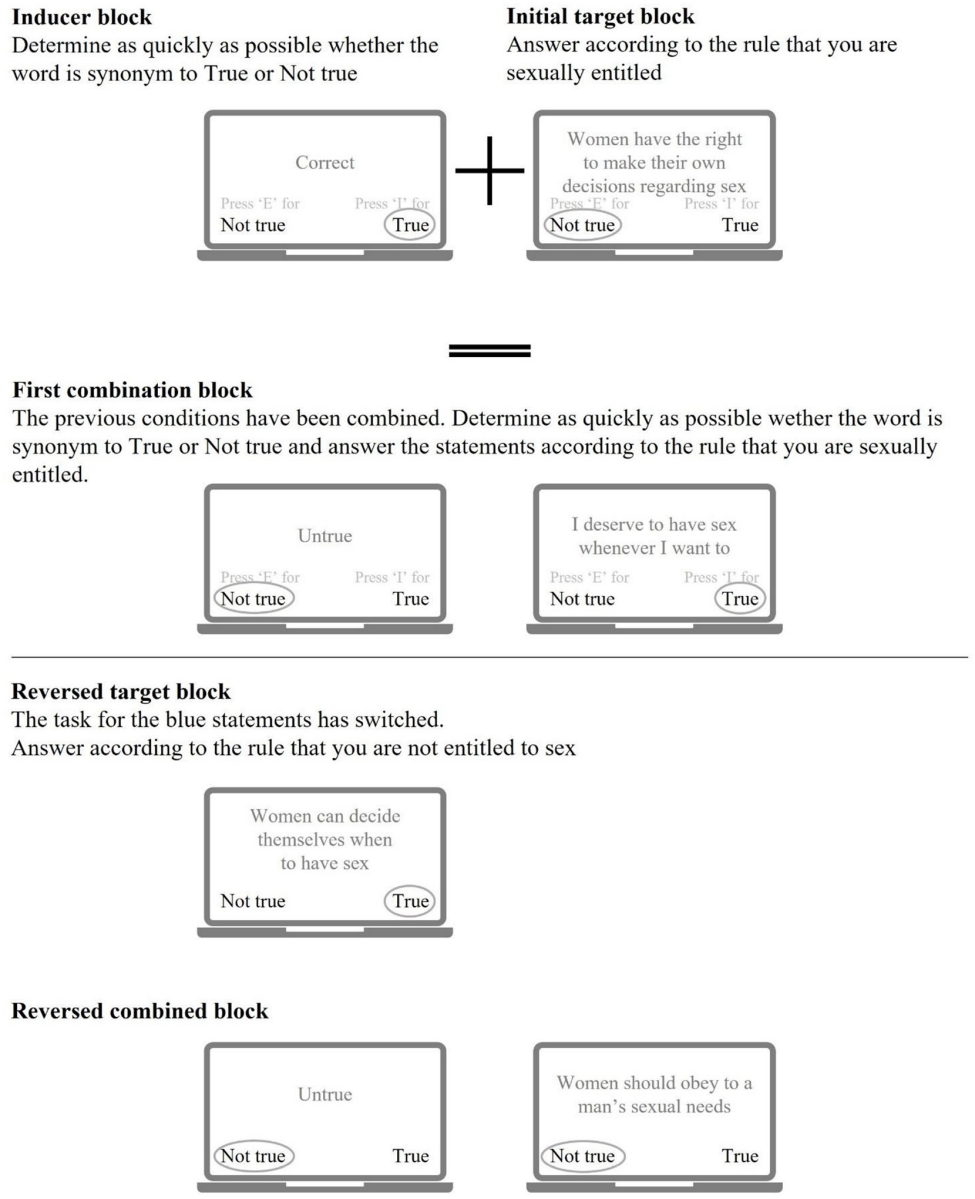
Schematic Picture of the Relational Response Task (RRT) Procedure.

As both the IAT and the IRAP have their limitations and strengths, a third measure was developed combining the positive qualities of the IAT and the IRAP; the Relational Responding Task (RRT; De Houwer et al., 2015). For a schematic picture of the RRT, see Figure 3. In the RRT, participants respond to statements following a specific rule/belief that is reversed between the test blocks—similar to the IRAP. Unlike the IRAP, this belief is always explicitly stated. Moreover, rather than changing the physical location of response keys, the RRT uses inducer trials on which stimuli are presented that refer to the concepts “true” or “false.” By including these inducer trials, the RRT has a similar, but simpler task structure—similar to the IAT—in which participants are asked to assign stimuli to categories in a way that varies across blocks. Combining the positive features of both the IAT and the IRAP, it is believed that the RRT is easier to administer, with relatively low error and dropout percentages yet should still be able to capture more complex implicit beliefs. To date, there are no published empirical studies, including the RRT to examine ITs related to sexual offending.

## Research Problem

To recap, indirect measures were designed to measure constructs on a more implicit level. Theoretically, it could be argued that indirect measures are ideal for measuring ITs because they represent a construct that is believed to be at a more “unconscious” level and might be influenced by several contextual factors (e.g., social desirability). However, it is unclear whether these indirect measures can be used validly with these more complex beliefs.

Furthermore, it has been argued that the IRAP is more suitable than the IAT in terms of measuring complex beliefs instead of cognitive associations and that the RRT is more suitable than the IRAP by measuring relations with a more straightforward task structure resembling the IAT. Nevertheless, no studies are comparing the performance of all three tasks and there is no evidence that the IRAP and RRT—both aimed at measuring more complex beliefs—empirically converge. The fact that both measures are classified as indirect does not guarantee that they measure the same construct, are influenced by the same psychological processes, share common variance, or have the same predictive abilities.

New alternatives to measure ITs seem promising, at least theoretically, but several questions remain. First, the IRAP and the RRT are considered to be cognitively demanding tasks, which might challenge the feasibility when used with specific populations in the forensic field (e.g., those with low intelligence, impulsiveness, attention deficits, etc.). Second, the feasibility, reliability, and concurrent, discriminant, predictive, and incremental validity of the IRAP and RRT in measuring cognitively complex beliefs has not yet been established and direct comparisons between these indirect measures do not yet exist.

## The Current Study

To address the open questions in the literature on the indirect assessment of ITs, the current study aimed to investigate the feasibility, reliability, and validity of the IAT, IRAP, and RRT. Because the investigated methods are rather novel in the field of sexual offending, their performance was evaluated not only when measuring ITs but also when evaluating relatively simple self-behavior associations related to the forensic field (i.e., aggressiveness). Such self-behavior associations (e.g., “*I hit*”) require less introspective ability and are less cognitively complicated and demanding. As for the indirect measurement of ITs, the IT Sexual Entitlement was chosen (e.g., “*I deserve to have sex whenever I need it*,” “*Women should obey a man’s sexual needs*”) as sexual entitlement is a construct that often endorsed in population samples (Widman & McNulty, 2010).

To assess feasibility, we investigated mean latency, error rates, and passing criteria—both in terms of their mean levels and their associations with age and education. Because of the potential cognitive burden of participation for forensic patients (i.e., the indirect measure and the duration of the study), the current study was conducted with a general population sample. We reasoned that if participants from the general population cannot perform on the IRAP and RRT in terms of passing criteria, error rates, and response latency, using such assessments in clinical or forensic populations would be problematic. For example, many forensic patients lack the cognitive flexibility, attentional capacities, and intelligence that may be required for these assessments (e.g., Hayes et al., 2001; Young & Cocallis, 2019). To assess the reliability of the various indirect measures, the internal consistency will be assessed as conclusions about individual scores are warranted without strong internal consistency.

Finally, to establish construct validity, we compared several indirect and direct measures (IRAP, RRT, IAT, self-reports, and other-reports) of two traits (i.e., Aggression and Sexual entitlement). This made it possible to investigate convergent and discriminant validity using a multitrait-multimethod approach (MTMM; see Campbell & Fiske, 1959). Using MTMM, monotrait-heteromethod (same trait but different method) associations would be interpreted as an indication of convergent validity. However, research has shown that direct and indirect measures (even when assessing the same topic) are only weakly related as they are suggested to assess distinct but related constructs (e.g., Fazio, 2007; Gawronski & Bodenhausen, 2006; Nosek & Smyth, 2007). Associations between direct and indirect measures are therefore expected to be positive, but the effect size will be quite small (Cohen, 1988, 1992). Strong associations are not expected because direct measures are influenced by deliberate responding, making them not straightforward validation criteria for indirect measures. Therefore, as a second criterion, we expected that the associations between different indirect measures would be higher than associations with direct measures (e.g., Ranganath et al., 2008). To consider discriminant validity, monotrait–heteromethod associations should be higher than the associations that have neither method nor trait in common (heterotrait–heteromethod). In addition, to complete the MTMM approach, it will be tested if trait factors are stronger than method factors, therefore the association between monotrait–heteromethod should be higher than heterotrait–monomethod associations. Moreover, the same pattern of relationships should be found for the heterotrait correlations in blocks measuring associations between the same method of measurement and associations between different methods.

Lastly, predictive and incremental validity was explored as the ability of indirect measure(s) to explain relevant outcome measures (i.e., the informant reported aggression and sexually aggressive behavior), over and above standard self-report measures.

## Method

### Participants

The total sample consisted of 111 male participants from the general population. Because sexual entitlement takes women as a reference, two participants were removed from the study because they reported an exclusive same-gender sexual preference. The final sample thus consisted of 109 male participants with an average age of 32.57 years (*SD*= 15.69; range 18–80 years; one participant did not report age correctly). Almost all participants (95.4%) reported having Dutch nationality and 17.4% had a migration background. Regarding the level of education, a small group (0.9%) only completed elementary school; 32.1% held a high school degree, 25.7% held a lower vocational degree; 24.8% held a higher vocational degree, and 16.5% held a university degree.

### Measurements

#### Direct Measurements

##### Sexual Entitlement Subscale of the Sexual Narcissism Scale

Sexual entitlement was measured using the 5-item Sexual Entitlement Subscale of the Sexual Narcissism Scale (SNS; Widman & McNulty, 2010). Items (e.g., “I feel I deserve sexual activity when I am in the mood for it”) are scored on a 5-point Likert-type scale (1 = *strongly disagree*, 5 = *strongly agree*), with higher scores indicating feeling more sexually entitled. Internal consistency of the subscale was found to be acceptable to good in other studies (α > .76; Noteborn et al., 2024; Widman & McNulty, 2010). In the current study, the sexual entitlement subscale’s internal consistency was somewhat lower, α = .65.

##### The Aggression Questionnaire

Self-reported aggression was measured using an adapted 17-item version of the Aggression Questionnaire (AQ; Buss & Perry, 1992; Dutch translation: Meesters et al., 1996). The items that were used describe aggressive behavior that is observable by others (cf. Banse et al., 2014; Noteborn et al., 2024). Each item was answered on a five-point Likert-type scale (1 = *extremely like me*, 5 = *extremely unlike me*). The 17-item adapted version of the AQ scale showed good internal consistency in this study (α = .82).

##### Sexual Experience Survey–Tactics First Revised Short Form

Sexually aggressive behavior was measured using the revised short version of the Sexual Experience Survey using the tactics first subscale (Sexual Experience Survey -Tactics First Revised Short Form [SES-TFR-SF]) (Abbey et al., 2005). For this study, all items were translated into Dutch. A back translation confirmed that the Dutch translations corresponded with the original English items. The SES-TFR-SF asks participants to report whether they, since the age of 14 years, have used (a) arguments and pressure, (b) lies or false promises, (c) invoking guilt or displeasure, (d) giving drugs or alcohol, (e) taking advantage when a woman is incapacitated due to drugs or alcohol, and (f) using physical force to engage in any sexual behaviors. Abbey et al. (2005) found that by first asking about tactics instead of the kind of sexual behavior (e.g., “have you ever overwhelmed a woman with continual arguments and pressure although she indicated she didn’t want to, to . . .” followed by kind of sexual behaviors vs. “have you ever fondled, kissed, or sexually touched a woman without her consent..” followed by the tactics), men were more likely to respond affirmatively. Each item was answered dichotomous (yes/no). If a participant had ever conducted any form of sexually aggressive behavior (i.e., answered “yes” on one or more items), he received a score of “1.” If a participant answered “no” on all items, he received a score of “0” indicating that he never conducted any form of sexually aggressive behavior. In total, 33 participants indicated to have behaved sexually aggressively (*Mnumber of sexually aggressive behaviors* = 2.36, *SD* = 1.80).

##### The Short Version of the Balanced Inventory of Desirable Responding

Social desirability (SDR) was measured using the dichotomous Dutch short version of the BIDR-6 (BIDR-20; Noteborn et al., 2024; Paulhus, 1984). The BIDR-D20 consists of 20 items measuring Impression Management (IM: 10 items) and Self-Deception Enhancement (SDE: 10 items) as two distinct dimensions of SDR. The IM and SDE scale has five positively keyed items and five negatively keyed items that are reverse scored before calculating the overall score. If respondents endorse a high number of IM statements, they may be intentionally tailoring their responses to impress the user with the questionnaire results (e.g., “When I hear people talking privately, I avoid listening”). Participants scoring high on SDE items are thought to report unrealistic, yet honestly believed, positive self-description (e.g., “I am fully in control of my own fate”). Respondents rated their agreement on the BIDR items on a seven-point (1 = *totally disagree*, 4 = neutral, 7 = *totally agree*) Likert-type scale. The BIDR-D20 is scored dichotomously: After recoding, only scores on the high end of the scale are counted (6-7 = 1, others are 0). Internal consistency of the BIDR-D20 IM (α = .71) and SDE (α = .62) were comparable with previous studies on the original version (e.g., Li & Bagger, 2007).

#### Indirect Measurements

##### Implicit Association Test

An Implicit Association Test (IAT) was developed to measure aggression.^2^ The IAT consisted of five blocks. In an initial block of trials intended for target concept discrimination, two different concepts (“I” and “Others”) appeared on a screen, and subjects classified stimuli regarding concepts as soon as possible by pressing one of two keys (i.e., response keys “e” for left and “i” for right which will be different in each indirect task [IAT, IRAP, RRT] to accentuate the fact that these are different tasks) into one of the concepts. Next, stimuli were presented on the screen and coupled with another pair of opposing concepts (e.g., words representing positive and negative valence; Aggression vs. Peaceful) using the same two keys (associated attribute discrimination block). In the third block, examples of all four concepts were classified, each being assigned to the same key as in the first two blocks (20 trials; e.g., I and Aggressive vs. Others and Peaceful). In the fourth block, the respondent was presented with the stimuli from the first block, but the response keys were switched (e.g., e = “I” and i = “Others” became e = “Others” vs. i= “I”). This was done so that the participant learns a reversal of response encoding for the target discrimination (reversed target concept discrimination block). The final fifth block was the same as in Block 3, with the only difference being that the target discrimination changed the key as learned in Block 4. The target was presented in white, while the attribute was presented in green to highlight the changes.

##### The Implicit Relational Assessment Procedure

Participants were first presented with practice bocks. The first block of trials in the consistent condition asked participants to respond in line with beliefs that were considered relationally consistent (i.e., being aggressive or sexually entitled; response keys “x” for left and “m” for right). What is considered relationally consistent is determined by the target detection group, that is, the focus of the measurement. Hence, when attempting to measure sexual entitlement and aggressive behavior, these concepts are considered relationally consistent. Therefore, what is deemed relational consistent does not necessarily reflect the consistency of the participant’s beliefs. The second block of trials in the inconsistent condition asked participants to respond in line with beliefs deemed relationally inconsistent with the focal construct (i.e., others being aggressive or not being sexually entitled). After each practice block, feedback was provided on the screen in the form of a percentage of correct responses and the average response latency for that block. The six test blocks (each 24 trials) that followed the practice blocks alternated between consistent and inconsistent (i.e., Test Block 1 with consistent trials, Test Block 2 with inconsistent trials, etc. until Block 6 with inconsistent trials).

##### The Relational Responding Task

Similar to the IRAP, the RRT required that participants respond according to specific beliefs. The RRT consisted of five blocks. The first RRT block included 20 inducer trials in which participants classified synonyms of True and False. In the second block (40 trials), participants were shown statements that they had to categorize as True (S on the keyboard) or False (Numpad 5 on the keyboard) by responding according to a specific rule/belief stated at the beginning of the block (e.g., please respond as if you are sexually entitled and that women have to obey a men’s sexual needs; You are aggressive and others are peaceful). The participant then had to indicate whether the statement was in line with the focal belief or not. In the first combined block, block three (80 trials), the principle of the first (Inducer trials), and second blocks (respond as if..) were combined using the same belief. The fourth block (40) was the same as the second, but the rule for responding was reversed (e.g., please respond as if you are not sexually entitled and women can make their own decisions regarding sex; You are peaceful, and others are aggressive). The final block (80 trials) combined the first and the fourth blocks. In line with the original RRT developed by De Houwer et al. (2015), we programmed the color of the stimuli to help participants switch between the inducer trials (orange) and the target trials (blue).

For all three measures, after instructions, the screen was cleared for 2,000 ms before the first trials started. By selecting the correct response in the corresponding block, the screen was cleared 750 ms before the next trial was presented. All stimuli remained visible until the participant pressed one of the response keys. If an incorrect response was selected, a red X appeared on the screen for 200 ms (directly below the stimuli) until the participant selected the correct response.

While it is recommended to counterbalance the test blocks to avoid block-order effects, it may distort correlations with direct measures and therefore confound individual differences in associative and relational strength (see Gawronski, 2002; Gawronski et al., 2011). For this reason, we decided not to counterbalance, and each participant started with the test block that was congruent/consistent with the focal constructs (I am aggressive/I am sexually entitled). All stimuli were presented using the font Ariel in bold with a height of 4.1% of the height of a 24-inch black screen.

#### Scoring Procedure of the Indirect Measures

During testing, no response latency cut-off was set for the IRAP. The use of sentences in combination with the possible complexity of the concepts made it impossible to determine an appropriate cutoff in advance. Therefore, regardless of percentage error or mean latency during the practice rounds, participants were allowed to proceed to the testing phase. This allowed us to calculate the attrition rate without any ceiling effects. Post hoc cutoffs were tested for 80% correct responses and various latency cutoffs. As suggested with the use of sentences, we opted for a cut-off of 3,000ms (Barnes-Holmes et al., 2010).

We adjusted the latency cut-offs of the IRAP for the average sentence length of the stimuli, as this differed between aggression (average of 2.8 words) and sexual entitlement (average of 12 words) stimuli. A meta-analysis (Brysbaert, 2019) indicated an average reading speed of 245 words per minute for the Dutch language. To translate this into single sentences and the response speed framework, we decided to divide the average word speed by 10, which resulted in an additional time of 68 ms for aggression and 291 ms for sexual entitlement (i.e., above the 3,000 ms).

For interested readers, we also implemented the standard of 2,000 ms and the cut-off of 5,000 ms, as used by Dawson and colleagues for the IT Children as Sexual Beings (Dawson et al., 2009). Results can be found in the online supplementary material. For all indirect measures, the strength of an association (IAT) or propositional relations between concepts (IRAP and RRT) was assessed by the *D*(1) measure, operationalized as the difference between the mean response latencies of congruent and incongruent test blocks, and divided by the pooled standard deviation of the response latencies (Greenwald et al., 2003). For the IRAP, the *D*(1) scoring procedure was modified such that besides an overall *D*(1) score, separate scores for the different trial types can be calculated (for more information see Barnes-Holmes et al., 2010). For all indirect measures, positive *D* values are indicative of self-associating as aggressive or sexually entitled.

Before calculating the *D*(1) measure for all three measures, participants with mean error rates > 2.5 standard deviations above the mean error rate were removed. Following Greenwald’s *D*(1) procedure, trials with response latencies >10,000 ms, and data from participants with more than 10% of the latencies being <300 ms were discarded.

### Procedure

Students completing their Bachelor’s in Psychology were asked to hand out an information letter to family and acquaintances informing them about the study and recruiting them as possible participants. Participants were given 2 weeks to consider their participation. Due to the sensitive nature of the topics, participants were told that the students were not involved in the study, except for the recruitment, and therefore would not have access to the data. Besides, it was made explicit that refusal to participate would have no consequence for the student. In the lab, participants signed an informed consent form and participated voluntarily. Participants were asked to fill out the self-report questionnaires (i.e., the SNS, 17-item AQ, and BIDR-D20) and complete five computer tasks (IAT, IRAP, and RRT for aggression, and IRAP and RRT for sexual entitlement). For all conditions and in line with previous studies, direct measures were presented before the corresponding indirect measure, as research has shown that presenting a participant with propositions (i.e., priming) increases the temporary activation level of the association in memory (e.g., Gregg et al., 2006). The order of presentation of the concept (i.e., aggression and sexual entitlement) and indirect measures were counterbalanced.

In addition, participants were asked to list the email addresses of family members, friends, or other members of their informal social network (with a maximum of five informants per participant). Listed informants were subsequently invited to fill out the adjusted AQ online All informants received a personalized email referring to the participant by name, asking to rate the participant’s aggression and explicitly informing them that their responses would not be shared with the participants. The internal consistency of the aggregate informant ratings (α = .91) was similar to those in other studies (Banse et al., 2014). Of the total sample of 109 male participants, 98 participants had one or more informant reports regarding aggression, with a total of 279 informant reports (*M*= 2.85 reports per participant, *SD*= 1.36; range 1–5). Participants who received informant reports were significantly younger (*M*= 31.43, *SD*= 42.64 vs. *M*= 42.64, *SD*= 24.18), *t*(106) = −2.289, *p* <.05, and less likely to report low vocational education as highest education (*χ*^2^ = 9.00, *p* <.001) than the participants who received no informant reports. There were no significant differences between those with and without informant reports in direct and indirect measures of aggression (*p*s <.05). Informants (46.2% male) reported varied types of relationships with the participant (e.g., grandfather, father, neighbor, friend, partner) and were on average 34.49 years old (*SD*= 16.53; 17–87 years).

The study was approved by the School of Social and Behavioral Sciences Ethics Review Board of Tilburg University (EC-2016.39). Although research has indicated minimal emotional harm in asking participants from the general population to report on possible sexually aggressive behavior and thoughts in the past (e.g., Edwards et al., 2012; Shorey et al., 2011; Yeater et al., 2012), besides the standard requirements (e.g., possibility withdrawal) several safeguards were put in place (discouragement for participation in case of a history of sexual abuse victimization, the possibility of emotional discomfort [i.e., the topic of the study] disclosed, the opportunity to consult a psychologist independent from the study). To inform participants, and in case participants were confronted with having deviant feelings or having displayed aggressive sexual behavior, participants were handed information about sexually aggressive behavior and thoughts and treatment for victims and offenders, including contact information after completing the study. While several steps were taken to safeguard the participants, no negative consequences were mentioned by the participants or observed by the researcher. The additional safeguard measures were not used.

### Statistical Analyses

To assess the feasibility of the IAT, IRAP, and RRT, data inclusion cut-offs were evaluated and compared. We investigated the extent to which participants who did not meet the inclusion criteria for the indirect measure differed in age and educational background from those who remained in the study. We also investigated the effect of age and education on mean latencies and error percentages. Mean differences between indirect measures in terms of mean latency and error percentage were examined using paired sample *t* tests and estimated effect sizes using Cohen’s *d.* Internal consistency of the indirect measures was calculated using split-half reliability with Spearman-Brown correction. Correlations between indirect and direct measures and the constructs’ aggression and sexual entitlement were structured along a Multitrait-Multimethod matrix (MTMM; Campbell & Fiske, 1959) to investigate convergent and discriminant validity. While the main focus of the MTMM will be on the expected pattern in associations, differences in associations were tested for significance using Fisher’s *r*-to-*z* transformations as additional support for validity. However, it was impossible to analyze all correlations within the MTMM framework because the IAT was applied only with one trait (i.e., aggression). Further correlational analyses outside the MTMM framework were performed to investigate associations between *D-score*s of all measures, traits and direct measures, and personal factors such as age and social desirability. Finally, linear regression analyses were performed to investigate the predictive and incremental validity of the indirect measures over and above the direct measurements of aggression and sexual entitlement. In all analyses, the first step included the main effects of the direct measures. In the second step, we added the main effects of the indirect measures to investigate to what extent the indirect measures added value over and above the direct measures. Bootstrap analyses (*n* = 1,000) were used to compute confidence intervals and robust estimates of standard errors due to the non-normal distribution of self-reported aggression. Coefficients were deemed significant when zero was not included in the 95% confidence intervals. As an estimate of effect size, squared semi-partial correlations (*sr*^2^) were calculated for significant effects. To examine the associations between Sexual entitlement IRAP and RRT, self-reported sexual entitlement, and self-reported sexually aggressive behavior (a dichotomous outcome), we used logistic regression analyses. This study was not preregistered. Materials and analysis code for this study are available by emailing the corresponding author.

## Results

### Feasibility

Of the three indirect measures (i.e., IAT, IRAP, and RRT), participants had the most difficulty passing the IRAP, especially for sexual entitlement (Table 1). In comparison, the dropout rates for the RRT and IAT were relatively low and comparable. The dropout rates for sexual entitlement were higher than for aggression, and most apparent for the IRAP. For the RRT and the IRAP, mean latencies and error rates were in the same range—albeit somewhat lower for the RRT—for both aggression and sexual entitlement, except for the higher error rate for the Aggression RRT. As expected, mean latencies and error rates were lowest for the IAT because of using one-word stimuli (Table 2). Mean latencies were higher for sexual entitlement than for aggression, whereas participants made more errors on the aggression tasks. In general, participants who passed the inclusion criteria for the IRAP also passed those for the RRT and IAT. Concerning the IAT and the Aggression RRT, two participants who failed the RRT passed the IAT, while one participant failed the IAT but passed the RRT.

**Table 2:** Mean Latencies and Error Rates and the Effects of Age and Education for All Indirect Measures of Aggression and Sexual Entitlement.

			IRAP		RRT		IAT
Variable			Aggression	Sexual entitlement	Aggression	Sexual entitlement	Aggression
		*n*	91	81	106	104	107
Mean latency (ms)			1,537.63	2,106.61	1,372.69	2,063.49	971.28
(*SD*; Range)			(349.65; 835.14–2,538.47)	(404.61; 1,231.82–2,978.91)	(366.10; 797.34–2,901.11)	(565.22; 1,078.31–3,917.13)	(225.40; 529.34–1,752.90)
Age		*r*	**.48**	.25	**.42**	**.21**	**.57**
Education			**χ** ^2^ **(3) = 13.66**	**χ** ^2^ **(3) = 10.81**	**χ** ^2^ **(3) = 25.38**	**χ** ^2^ **(3) = 10.47**	**χ** ^2^ **(3) = 16.56**
1	vs.	2	***U* = 177**, *z* **= −2.69**, *r* **= .37**	***U* = 129**, *z* **= −2.79**, *r* **= .41**	***U* = 185**, *z* **= −3.79**, *r* **= .49**	***U* = 227**, *z* **= −2.86**, *r* **= .38**	***U* = 248**, *z* **= −3.02**, *r* **= .39**
1	vs.	3	***U* = 186**, *z* **= −3.28**, *r* **= .44**	*U* = 176, *z* = −2.24	***U* = 155**, *z* **= −4.51**, *r* **= .57**	*U* = 289, *z* = −2.47	***U* = 217**, *z* **= −3.63**, *r* **= .46**
1	vs.	4	*U* = 180, *z* = −1.37	*U* = 188, *z* = −0.89	*U* = 193, *z* = −2.29	*U* = 280, *z* = −0.50	*U* = 239, *z* = −1.43
2	vs.	3	*U* = 216, *z* = −0.57	*U* = 128, *z* = −1.06	*U* = 290, *z* = −0.87	*U* = 315, *z* = −0.17	*U* = 322, *z* = −0.52
2	vs.	4	*U* = 112, *z* = −1.27	*U* = 74, *z* = −2.02	*U* = 189, *z* = −0.89	*U* = 153, *z* = −1.60	*U* = 178, *z* = −1.34
3	vs.	4	*U* = 125, *z* = −1.59	*U* = 106, *z* = −1.27	*U* = 157, *z* = −1.99	*U* = 177, *z* = −1.53	*U* = 159, *z* = −1.95
% Error (*SD*)			5.46 (2.93)	4.36 (2.95)	7.89 (5.57)	4.40 (4.01)	3.39 (2.84)
(Range)			(0.69–13.89)	(0.00–13.19)	(0.00–26.25)	(0.00–17.50)	(0.00–13.13)
Age		*r*	**−.27**	−.16	−.13	**−.22**	−.15
Education			χ^2^(3) = 3.21	χ^2^(3) = 0.81	χ^2^(3) = 1.65	χ^2^(3) = 2.41	χ^2^(3) = 3.31

*Note.* Education 1 = High School; Education 2 = Low vocational education; Education 3 = High vocational education; Education 4 = University. One participant did not finish the RRT Entitlement due to time constrains, therefore the total number of participants is 108 instead of 109. Because one participant did not indicate age correctly, he was not included in the analyses (*N* = *N* − 1). Sign. values are in bold.

To shed more light on dropouts, we compared age and educational differences between (a) participants who passed and failed the criteria for the IRAP and (b) participants who passed the RRT or the IAT vs. those who failed the IRAP.

Across comparisons, there were no significant differences in age or education between participants who passed or failed the passing criteria of the IRAP for either Aggression or Entitlement. In addition, there were no significant differences regarding age or education between participants who passed the RRT or the IAT versus those who failed the IRAP. For a complete overview of the differences between conditions, see the online supplementary material.

The effects of age and education level on the mean latency and error percentage can be found in Table 2. Small to medium associations between higher mean latencies and being older were found for all measures, except for sexual entitlement assessed with the IRAP. Being older was negatively associated with the error percentage for the Aggression IRAP and the Sexual Entitlement RRT. Regarding education, no differences in error percentage were found (see Table 2). However, mean latencies differed based on educational level. Overall, participants who completed high school were significantly faster than participants who completed vocational education (effect size *r* .37–49). Participants who completed high school were also significantly faster than participants who completed higher vocational education (effect size *r* .44–57); however, only for Aggression. Median values for each group can be found in the online supplementary material.

Differences in mean latency and error percentage between indirect measures can be found in Table 3. Comparisons between the indirect measures showed those mean latencies were significantly higher for the IRAP and lower for the IAT. The Aggression RRT had the highest error rates, though there was no significant difference in error rates for sexual entitlement. Error rates of the IAT were significantly lower than the IRAP and RRT error rates. Mean latencies and error rates were significantly and positively associated within the same trait (mean latency *r* = .68–.84; percentage error *r* = .40–.57) and across methods and traits (mean latency *r* = .39–.78; percentage error *r* = .31–.50). However, the Aggression and Sexual Entitlement RRT error rates only showed a small correlation (*r* = .21).

**Table 3:** Mean Differences in Mean Latency and Error Rate Between the Indirect Measures.

		Aggression (*N* = 91)			Sexual entitlement (*N* = 80)	
Condition		IRAP	RRT	IAT	IRAP	RRT
Mean latency in ms	*M* (*SD*)	1,537.63 (349.65)	1,342.03 (323.29)	954.22 (209.62)	2,109.53 (406.30)	1,910.12 (446.15)
	*t*	*t(*90) = 9.601** *d* = 1.01			*t*(79) = 6.639** *d* = 0.74	
			*t*(90) = 15.262** *d* = 1.60			
		*t*(90) = 23.674** *d* = 2.48				
% Error	*M* (*SD*)	5.46 (2.93)	7.48 (5.40)	3.04 (2.59)	4.30 (2.90)	4.02 (3.54)
	*t*	*t*(90) = −4.320** *d* = 0.45			*t*(79) = .771	
			*t*(90) = 8.654** *d* = 0.91			
		*t*(90) = 7.604** *d* = 0.80				

** Correlation is significant at the .001 level (2-tailed).

To directly compare the indirect measures, participants who completed all indirect measures for either aggression (*n* = 91) or sexual entitlement (*n* = 80) were included in the following analyses.

### Descriptives and Reliability

**Figure 4.**
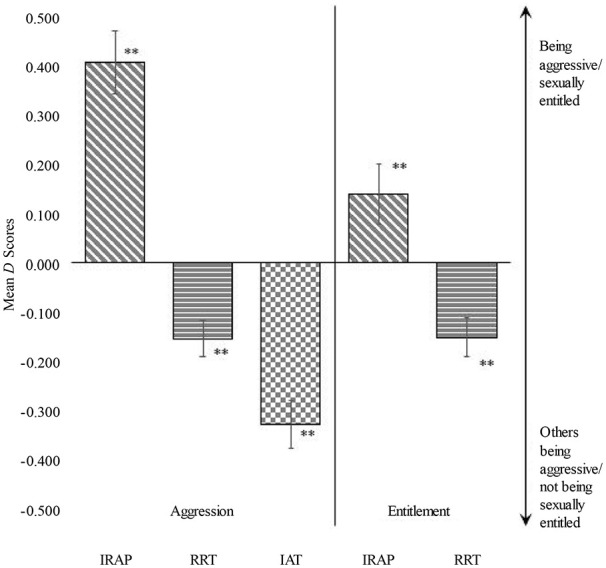
Mean D-Scores of the IRAP, RRT and IAT for Aggression and Sexual Entitlement.

Mean *D-score*s can be found in Figure 4. All means were significantly different from zero. As it concerns a general population sample, we expected that participants would, on average, identify less with sexual entitlement and aggression, thus suggesting negative *D-score*s. The *D-score*s of the RRT and IAT were in line with this expectation (i.e, negative *D-score*s). However, IRAP *D-score*s were positive, indicating that on average, participants would be considered self-associating as aggressive or sexually entitled.

We also compared pairs of individual *D-score*s between tests and computed a version of the reliable change index to indicate significance. For aggression, 8.8% to 24.2% had a significantly different *D-score* when comparing the IRAP with the RRT, 16.5% to 29.7% had a significantly different score when comparing the IRAP with the IAT.^3^ Finally, 14.3% o 23.1% of the cases had a significantly different *D-score* on the RRT and the IAT. For sexual entitlement, 17.5% to 18.8% of the participants had significantly different scores on the IRAP and the RRT.

Reliability coefficients are presented in Table 4. Although low, internal consistencies are in line with previous research concerning indirect measures (e.g., De Houwer et al., 2015; Nosek et al., 2007). Note, however, that the internal consistency for the Aggression IRAP was extremely low (α = .28), and caution is warranted when interpreting the following results.

**Table 4:** Multitrait-Multimethod Matrix Direct and Indirect Measures of Aggression and Sexual Entitlement and Additional Correlations Between Study Variables.

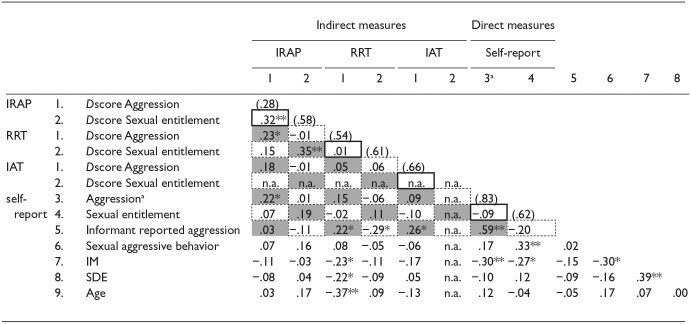

*Note*. MTMM can be found in the upper part of the table. Monotrait–heteromethod correlations (validity diagonals) are presented in gray. Reliability diagonals are the values in parentheses. Internal consistency for the indirect measures is calculated using split-half reliability with Spearman brown correction. Internal consistency for the other measures is calculated using Cronbach’s alpha. Each heterotrait–monomethod correlation is enclosed by a solid line. Each heterotrait–heteromethod correlation is enclosed by a broken line. Monomethod blocks consist of the Reliability diagonals and the heterotrait–monomethod correlations. Heteromethod blocks consist of Monotrait–heteromethod correlations and heterotrait–heteromethod correlations. Sexual aggressive behavior is a dummy variable with having shown sexually aggressive behavior serving as the reference group. **Correlation is significant at the .001 level (2-tailed). *Correlation is significant at the .05 level (2-tailed). *N* differed per analyses. For Aggression *n* = 91; For Entitlement *n* = 80; n for both Entitlement and Aggression combined was 76. For correlations between self-reported measures highest sample size was chosen (i.e., 91). For exact sample sizes used see the appendix. For a clearer visual overview of the MTMM analyses, the variable Sexual entitlement IAT was included in the table.

a Due to non-normal distribution Spearman Rho was calculated.

### Validity

#### Multitrait–Multimethod Matrix

Multitrait ***–*** multimethod matrix results are presented in Table 4. Only for the Aggression IRAP, the reliability value was not consistently the highest correlation, indicating that the IRAPs correlation with itself was lower than the correlation with other measures (i.e., Sexual Entitlement IRAP). Concerning convergent validity, monotrait–heteromethod correlations indicated that the IRAP and RRT were positively associated with both aggression and sexual entitlement. Examining the associations between direct and indirect measures, being more aggressive, as indicated by the IRAP, was significantly associated with higher self-reported aggression. However, the associations between the Aggression IRAP and RRT, and the Aggression IRAP and self-reported aggression were similar in size and direction. When considering the association between informant-reported aggression and the aggression IRAP RRT and IAT, a higher score on the RRT and the IAT were both associated with a higher level of aggression reported by the informants. The expected trend for convergent validity was found for the RRT, the Sexual Entitlement IRAP, and the corresponding self-report measures. However, it is important to note that these associations were non-significant and not significantly higher than the correlation between the indirect measures (Fisher’s *r*-to-*z* transformations *p*s > .05).

Supporting discriminant validity, for the RRT, the IRAP, and the self-report measures, monotrait–heteromethod correlations were higher than the heterotrait–heteromethod correlations. For the IAT, however, monotrait–heteromethod correlations with the RRT and the direct measures were not higher than heterotrait-heteromethod correlations. It should be noted that all correlations were close to zero. The monotrait–heteromethod correlations with the IRAP were higher than heterotrait–heteromethod correlations. More importantly, associations indicated a method factor for the IRAP as the heterotrait–monomethod correlations mostly exceeded the monotrait–heteromethod correlations. However, almost all differences in associations were not significant (Fisher’s *r*-to-*z* transformations *p*s > .05). Finally, associations between different methods and different traits were low as expected.^4^

Moving to correlations with the *D-score*s, analyses indicated that higher *D-score*s on the Aggression RRT and IAT were significantly associated with higher levels of informant-reported aggression, though correlations were small. Self-reported aggression and informant-reported aggression were positively associated. Concerning social desirability, Aggression RRT *D-score*s had a small association with lower levels of IM and SDE. Scores on direct assessments (self-reported aggression, sexual entitlement, and sexually aggressive behavior) were also associated with lower levels of IM. When considering age, only for the Aggression RRT there was a moderate negative association, indicating that being younger was associated with higher levels of aggression measured with the Aggression RRT.

#### Predictive and Incremental Validity

Table 5 shows the results of the hierarchical regression analyses of informant-reported aggression on self-reported aggression and the *D-score*s of the Aggression IRAP, RRT, and IAT. The main effects indicated that more self-reported aggression (*sr*^2^ = .32) and higher levels of aggression indicated by the IAT *D-score*s (*sr*^2^ = .04) were associated with higher levels of informant-reported aggression. However, including the indirect measures did not significantly contribute to the explanation of informant-reported aggression over and above the use of self-reports (Δ*R*^2^ =.05, *p* >.05).

**Table 5:** Regression Analyses Direct and Indirect Measures of Aggression and Sexual Entitlement on Informant Reported Aggression and Sexual Aggressive Behavior.

	Informant reported aggression			Sexual aggressive behavior			
	B (*SE*)	95% CI		B (*SE*)	Wald	Exp (B)	95% CI for Exp(B)
Model 1			Model 1				
R^2^	**.38**		Nagelkerke R^2^	**.15**			
Constant	**0.85 (−0.18)**	**[0.46, 1.19]**	Constant	**−3.51 (1.02)**	**11.88**	**0.03**	
Aggression	**0.55 (0.08)**	**[0.40, 0.73]**	Sexual entitlement	**1.50 (0.53)**	**8.08**	**4.48**	**[1.59, 12.59]**
Model 2			Model 2				
ΔR^2^	.05		Nagelkerke R^2^	**.19**			
Constant	**1.02 (0.21)**	**[0.60, 1.42]**	Constant	**−3.86 (1.10)**	**12.36**	**0.02**	
Aggression	**0.53 (0.09)**	**[0.36, 0.72]**	Sexual entitlement	**1.50 (0.55)**	**7.47**	**4.48**	**[1.53, 13.14]**
*D*score IRAP	-0.04 (0.07)	[−0.18, 0.10]	*D*score IRAP	0.88 (0.71)	1.52	2.41	[0.60, 9.72]
*D*score RRT	0.11 (0.13)	[−0.15, 0.37]	*D*score RRT	−0.98 (0.83)	1.39	0.38	[0.07, 1.91]
*D*score IAT	**0.26 (0.12)**	**[0.04, 0.50]**					

*Note*. Sign. values are in Bold. Aggression *N* = 84; Sexual entitlement *N* = 80. As the both regression analyses indicated the presence of one multivariate outlier, sensitivity analyses were performed. The results did not differentiate.

Concerning sexual entitlement, logistic regression analyses indicated that higher levels of self-reported sexual entitlement were related to sexually aggressive behavior (odds ratio = 4.48). Adding the indirect measures to the model did not significantly add to the explanation of sexually aggressive behavior over and above the self-report measure.

As one of the claimed advantages of the IRAP is the ability to calculate several trial type scores, we also performed the MTMM and regression analyses for all trial types. Readers interested in the results of the sub-trials of the IRAP are referred to Online supplementary material.

## Discussion

The current study investigated the feasibility, reliability, and validity of the IAT, the IRAP, and the RRT in measuring ITs. Overall, none of the indirect measures ticked all boxes in terms of the aforementioned criteria. While the IAT had comparative methodological strengths (i.e., internal consistency, feasibility, predictive ability), the fact that it could not be used to measure beliefs—sexual entitlement—together with other interpretational problems, renders the measure not suitable for these purposes. When looking at the IRAP and RRT, the RRT—while having its weaknesses—outperformed the IRAP in terms of feasibility, reliability, and convergent validity in measuring behavior and cognition. The greatest difficulty with the use of the IRAP was its feasibility, the demonstrated influence of a method factor, and, in line with this, the possible extra cognitive challenge that comes with the task structure. The results will be discussed in further detail in the following sections.

### Feasibility

Because it is desirable to have an indirect measure that can be easily administered to a large and representative sample, we looked at the feasibility of the three indirect measures based on dropout rate and influential factors. In terms of feasibility, when looking at the results after applying a 3,000 ms threshold, participants had by far the most difficulties in completing the IRAP criteria, as evidenced by a dropout rate of 16.5 (aggression) and 25.7% (sexual entitlement). Although these dropout rates are problematic when using the IRAP in clinical practice, they are common in IRAP research (Drake et al., 2018; see overview Hughes & Barnes-Holmes, 2013). Dropout rates for the RRT were low and in line with previous research (De Houwer et al., 2015; Glashouwer et al., 2018; Heider et al., 2018). As expected, dropout rates were also low for the IAT.

When looking at factors that may influence feasibility, all three measures were influenced to some extent by external factors (age, education) when considering mean latency and error rate. In line with previous research (e.g., Nosek et al., 2007), these effects were no longer present when the compound *D-score* was included, except for the association between age and the Aggression RRT *D-score*. Nevertheless, this finding is consistent with research indicating that aggressive behavior decreases with age (e.g., Tremblay, 2010) and could therefore also be interpreted as a normative age trend.

Looking at the differences between the measurement of behavior and beliefs, average response latencies were all below or around 2,000 ms. Mean latencies for aggression were comparable to previous studies, whereas the latencies for sexual entitlement were slightly higher (e.g., Barnes-Holmes et al., 2008; De Houwer et al., 2015). Although mean latencies were higher for sexual entitlement than for aggression, on average participants made more errors on the aggression tasks. One explanation could be that participants needed more time to complete the Sexual Entitlement indirect measures due to the longer sentences. However, the longer latencies for sexual entitlement could also be because of the more complex and sensitive nature of the topic, as participants were more inclined to answer in line with social norms and therefore took longer to answer. Such a larger time window in turn might leave room for fewer errors. This may indicate that using the RRT and IRAP with sensitive topics approximates a more direct measure. In other words, allowing participants so much time to answer might give rise to response latencies that can no longer be considered automatic and “implicit” and which might instead reflect a more elaborate and explicit response.

### Reliability

The internal consistency of the Aggression IRAP can be considered unacceptable, although it is not uncommon with the IRAP to find such low values (e.g., Barnes-Holmes et al., 2011). Internal consistency for the Sexual Entitlement IRAP can be considered at sufficient levels for research purposes (e.g., DeVellis, 2003; Nunnally & Bernstein, 1994) but not for individual assessment (Bland & Altman, 1997). The low internal consistency in combination with the high dropout rate of the IRAP could indicate that the IRAP procedure is too difficult. Specifically, switching instructions between blocks and response keys between trials requires a high level of cognitive flexibility. The cognitive flexibility that is needed results in a higher dropout rate because not all participants are able to switch between blocks and trials. Whereas the ability to adapt to the specific blocks is also applicable to the IAT and the RRT, the additional switching of the response keys on a trial basis in the IRAP asks for extra cognitive effort.

In contrast, the internal consistency of the RRT, IAT, and Sexual Entitlement IRAP is acceptable for research purposes according to some researchers (De Houwer et al., 2015; DeVellis, 2003; Nosek et al., 2007), but insufficient for individual assessment in clinical practice (Bland & Altman, 1997). Researchers argue that for (experimental) research purposes these internal consistencies can be acceptable. However, low internal consistency does has an effect on research findings, as they for example attenuate correlation coefficients (e.g., Nunnally & Bernstein, 1994). The difference in internal consistency between the RRT and the IRAP for the construct aggression is surprising as the same items were used in both measures. Again, the additional cognitive challenge that is presented with the IRAP could explain these differences. Thus, a solution could be to reduce the difficulty of the items, for example by reducing the complexity of sentences. Another possibility is to increase the number of items and/or trials, although this would limit the feasibility of the measure. It also has to be mentioned that the internal consistency of these indirect measures is actually an indication of consistency in time, and therefore not directly comparable with the internal consistency of direct measures. In addition, most direct measures are answered on a Likert-type scale leaving a range of answering options, instead of being forced to a more black-and-white option under time constraints. Perhaps, as formulated, aggression is a more black-and-white option for participants, whereas sexual entitlement is more viewed as present or not. More research is needed to investigate these issues.

### Validity

To investigate the validity of indirect measures, we used four approaches. First, evidence for convergent validity would consist of significant correlations with alternative indirect measures, preferably measures that have been very well established. Because there is no indirect measure that can serve as a “gold standard” criterion, this type of validity is difficult to establish. Ideally, various indirect measures tap into similar processes, which would result in convergent correlations. Second, it could be argued that indirect measures should converge with direct measures. This is not straightforward as direct measures might be subject to self-representation biases, and one would expect that indirect measures would be able to better measure psychological constructs validly, lowering the association between the direct and indirect measures. Third, predictive validity could be established by investigating correlations with behavioral outcomes, preferably assessed independently from participant reports to avoid reported method bias. In the current study, this was done for aggression by asking informants to rate participants’ aggression levels. Although less optimal for sexual entitlement, participants were asked to rate their sexually aggressive behavior. Fourth, one could demonstrate discriminant validity by investigating associations with social desirability and the difference between different traits and measures. Evidence for discriminant validity would be obtained in case of low or absent correlations with social desirability and higher trait above method associations.

When evaluated in light of the types of validity outlined earlier, there was limited evidence for the validity of the IRAP. Although the IRAP converged with the RRT and predicted self-reported aggression, we found evidence for a method factor for the IRAP, indicating that the IRAP produced associations based on its procedure and not the measured construct. The association between the Aggression IRAP and the Sexual Entitlement IRAP was one of the highest associations found in this study. The research argues that this person-by-method interaction could be due to processing speed (e.g., Blanton et al., 2006) or executive functioning (Ito et al., 2015). However, the IRAP did not predict observed assessed behavior (lack of predictive validity), although it did demonstrate some discriminant validity in terms of independence from socially desirable responding.

Some evidence for validity was found for the RRT. Although the RRT did not correlate with the IAT, it correlated with the IRAP, perhaps reflecting a similarity in stimulus properties (i.e., both measures used the same sentence stimuli). More importantly, higher levels of seeing oneself as aggressive based on the RRT were associated with higher levels of informant-reported aggression, a more objective indicator of aggressiveness. Nonetheless, this association was insignificant after controlling for self-reported aggression. A possible threat to the discriminant validity of the RRT was that it was significantly correlated with social desirability. On the one hand, this could represent a social desirability bias, indicating that the RRT might be viewed as a more direct measure. On the contrary, research has favored the interpretation of social desirability as a substantive personality characteristic. This characteristic could reflect participants’ personality characteristics regarding the ability to adjust to social situations and seek approval from others (i.e., ‘interpersonally oriented self-control’; Uziel, 2010; see also Banse et al., 2014). However, if social desirability reflects an aspect of self-control, an association between informant-reported aggression and social desirability would also have been expected.

Turning to the IAT, based on the MTMM framework we found little evidence for convergent validity of the IAT, as there was no significant association with direct or indirect measures. One explanation could be that the underlying association between “Aggression” vs. “Peaceful” and “I” vs. “Me” does not represent the same meaning as the sentences used in the RRT, in which the underlying nature is clearer. As explained in the introduction, the association between only two constructs can be ambiguous. It may be that the IAT measures a different component of Aggression than the RRT and the IRAP as, for instance, “I am aggressive” and “I want to be aggressive” are in an IAT framework associative identical. Furthermore, it has to be mentioned that the IAT works with associative stimuli that should preferably not consist of negations. This often results, as is with the aggression IAT, in a comparison with, for instance, others instead of with the self. The problem with using Others as the associative discrimination group is that finding yourself aggressive and finding others aggressive are not mutually exclusive.

In terms of predictive validity, the IAT explained the score on informant-reported aggression, even though the IAT did not have added value over and above the use of self-report. Finally, because it was not possible to use the IAT to measure sexual entitlement, no clear statement about discriminant validity based on the MTMM framework can be made. However, the IAT demonstrated some degree of discriminant validity in that scores did not correlate with social desirability.

The low correlation between the indirect measures is not surprising when looking at the average compound *D-score*. In terms of compound scores, the indirect measures seemed to indicate opposite effects: While on average participants on the IRAP indicated themselves as aggressive and sexually entitled, on the RRT and the IAT participants saw others as more aggressive and did not consider themselves sexually entitled. On the individual level, the choice for an indirect measure in a clinical setting could thus affect the outcome in terms of assessment. In the current study, one out of five to six participants would receive a significantly different clinical classification depending on whether the IRAP or the RRT is used for sexual entitlement. While this difference might seem relatively small in terms of research, it is not clear which of the indirect measures assesses sexual entitlement most accurately. This notion alone warrants the use of these measures when conclusions are inferred from them in the clinical field. One could argue that the indirect measures could however be used as supportive measures of other indications of for instance sexual entitlement. Yet, the complexity of the measures, the rather lower feasibility, and the low internal consistency questions the extra time constraint as just a supportive measure.

Finally, it needs to be acknowledged that low correlations between indirect and direct measures—of either the same or a different construct—are expected if we generalize previous research in different fields, such as self-esteem, impulsivity, and racial and ethnic discrimination (e.g., Bar-Anan & Nosek, 2014; Cyders & Coskunpinar, 2011; Oswald et al., 2013). These low correlations could indicate that these measures tap into a (small) amount of common variance. However, the small effect sizes indicate that what is being assessed using the direct and indirect measures at hand indicates more variability than common variance. The question remains what this variability indicates. It could be argued that this indicates that different, mostly unrelated, constructs are being measured and could therefore indicate a degree of discriminant validity or perhaps give insight into the interplay between implicit and explicit cognitions (Nosek, 2005, 2007). Yet, it could be argued that the small amount of common variance indicates that different aspects of the construct are measured (e.g., Cyders & Coskunpinar, 2011) or that direct measures are influenced by deliberate evaluations and indirect measures by the procedure used (Bar-Anan & Nosek, 2014). Hence, although, performance on a task relates to some outcome of interest, it is not always clear that the underlying process the indirect measure represents corresponds to similar theoretical constructs or traits (e.g., Cyders & Coskunpinar, 2011).

### Limitations

This study has several limitations. To start, our power analysis (conducted using G*Power, Faul et al., 2009) assumed a medium effect size of f^2 = .15. This corresponds to a correlation coefficient of around .36, which is higher than correlations that are typically found when associating direct and indirect measures (e.g., Greenwald & Lai, 2020). Thus, our study might have been underpowered to detect certain validity correlations, although we think it was sufficiently powered to detect correlations between different indirect measures. In addition, we do not yet know much about the predictive validity of measures of implicit theories, therefore it was hard to establish which effect size should be assumed. However, power analyses indicate that a total number of *N* = 1,095 participants would be required to obtain an effect size of f^2 = .01 (i.e., *r* = .10), as usually found with studies looking at implicit-criterion associations. The protocol for the power analyses performed can be found in the online supplementary material.

Furthermore, whereas Campbell and Fiske (1959) explicitly recognized that one could have an incomplete MTMM design, this is not ideal. As it was not possible to measure sexual entitlement with the IAT, no further statement could be made about the IAT’s discriminant validity. However, the fact that it was not possible to measure a complex belief with the IAT of course renders this method invalid for these purposes. Furthermore, whereas aggression was measured with a reliable behavioral outcome measure, namely informant reports, sexually aggressive behavior was measured using self-report. This could raise concerns that shared reporter variance may have inflated the associations with other direct measures.

To be consistent with previous research suggestions (Gregg et al., 2006), direct measures were administered before the corresponding indirect measure, because research has indicated that presenting a participant with similar propositions increases the temporary activation level of the association in memory (i.e., priming). However, if the use of a self-report measure is necessary to properly access automatic representations, it would prolong testing time and thus possibly limit concentration and cooperation in forensic patients—something that is already limited. In addition, it could be that some people are more capable of making associations in memory, and therefore also faster in making these associations. To eliminate the priming effect and take a closer look at the capabilities of indirect measures, one should prolong the time between self-report and indirect measures.

Concerning the MTMM framework, the correlation between two measures that attempt to measure the same construct is influenced by the internal consistency of both measures (Nunnally & Bernstein, 1994, p. 214). As the internal consistency of all indirect measures was relatively weak, the associations were possibly an underestimation (see online supplementary material for disattenuated correlations). In addition, while the construct aggression was chosen due to its forensic relevance, it needs to be recognized that the chosen constructs aggression and sexual entitlement both fall under antagonistic traits. Therefore, one could argue that claiming discriminant validity using these constructs is debatable. On the contrary, antagonism is a broad spectrum of traits on one end of a domain dealing with an orientation toward others that runs from antagonism to agreeableness (Lynam & Miller, 2019). In addition, as support for discriminant validity, we did not only look at the correlation between the different constructs but also looked at the different correlations between the same trait using different methods as an extra criterion.

Another limitation is that we restricted the number of practice rounds of the IRAP to two instead of the maximum of six. Although it could be argued that increasing the number of practice rounds would result in lower dropout rates, increasing the number of practice rounds would further increase the time needed to complete the IRAP, which was already about 10 to 15 min. That is, increasing fluency might also increase fatigue. A possible solution would be to increase the practice round to meet the passing criteria, followed by a small break before moving on to the testing phase.

Finally, we did not set a cut-off for response latency during testing for the IRAP, as the use of sentences in combination with the possible complexity of the concepts made it impossible to a priori determine inclusion criteria. However, some research suggests lowering the error percentage to increase the passing rate (e.g., Timko et al., 2010; Vahey et al., 2009). Increasing mean latency in combination with lowering the error percentage could have resulted in a larger sample. However, this would question the indirectness and accuracy of the measure.

### Future Research

Further research is needed to further evaluate the feasibility, reliability, and validity of the indirect measures when assessing ITs or antisocial behavior in a larger sample. Foremost, whereas employing the RRT in a general sample seems feasible, the use of clinical samples is still an open question. On the one hand, it could be that indirect measures are better in measuring sexual entitlement when the proposed belief is more prevalent. On the contrary, indirect measures might be too complex for use in some clinical populations. More research should also be conducted to establish the validity of indirect measures in clinical populations. For example, criminal records could be used to measure sexually aggressive behavior, instead of self-reports, which would boost the validity of claims.

One shortcoming of all three indirect measures is their inability to measure several beliefs simultaneously. Specifically, when assessing ITs, the ability to measure all seven ITs in one indirect measure would be highly efficient. A recent study developed an indirect measure that allows for testing multiple ITs, called the Propositional Evaluation Paradigm (PEP; Müller & Rothermund, 2019). The PEP uses a sentence priming paradigm that presents statements that are either true or false in a word-by-word fashion. After the word-by-word presentation of the sentence (“I—am—sexually—entitled”), participants are prompted with the response word “true” or “false” and need to press the corresponding response key. Each sentence is shown with each response (e.g., “I—am—sexually—entitled”—“true” or “I—am—sexually—entitled”—“false”). The difference in reaction time between the two evaluations of the sentence is indicative of the extent to which a participant tends to evaluate the sentence. The PEP design allows for testing several ITs in the same task as the design does not require a participant to switch between instructions, which would also limit the cognitive flexibility that is needed. Whereas the first results of the PEP seem promising (Müller & Rothermund, 2019), further research is needed to see if the PEP could be a better alternative for the IRAP and RRT and whether it can be used with complex beliefs such as ITs of sexual offending.

### Applied and Clinical Implications

When looking at the results of the current study in light of applied and clinical implications, we believe several (related) questions need to be kept in mind when using indirect measures. First, is it feasible for the patient/target population to complete one of the indirect measures in terms of cognitive flexibility and drop-out rates? Second, which measures are most suitable? For example, the IAT was not suitable for measuring complex beliefs consisting of several associations. Third, what is being measured and how is the outcome related to the research or clinical purpose? There is much unclarity about what these measures or methods actually measure: beliefs that an individual may wish to conceal (Barnes-Holmes et al., 2006), beliefs that are activated automatically (Wilson et al., 2000), beliefs that an individual may not be able to identify introspectively (Greenwald & Banaji, 1995), or perhaps another underlying process. Furthermore, as the self-report measures in the current study showed some predictive validity, one could question whether the use of an indirect measure is needed and what they add to the outcome of interest.

## Conclusion

Overall, the performance of the indirect measures was somewhat disappointing, and no indirect measure emerged as a clear front-runner. Based on the feasibility, reliability, and validity, the RRT seems to be a somewhat better choice compared with the IRAP. Whereas the reliability and feasibility of the IRAP in the current study were limited, the RRT and IAT might be feasibly used in several populations. It has to be noted that for the IAT this is, in terms of behavioral concepts, not the assessment of the more complex phenomenon. However, while arguably acceptable for research purposes, the relative difference in clinical implications between these indirect measures, combined with the mixed associations with (other) measures, the question remains what is exactly being measured using these assessments. Self-report measures indicated better reliability and were better able to explain (sexual) aggressive behavior. The use of indirect measures in forensic settings—or any setting for that matter—therefore needs to await further research because the outcome of assessments can have real consequences for individuals and society.

## Supplemental Material

sj-docx-1-asm-10.1177_10731911241245009 – Supplemental material for Assessing Implicit Theories in Sexual Offending Using Indirect Measures: Feasibility, Reliability, and Incremental Validity Supplemental material, sj-docx-1-asm-10.1177_10731911241245009 for Assessing Implicit Theories in Sexual Offending Using Indirect Measures: Feasibility, Reliability, and Incremental Validity by Mirthe G. C. Noteborn, Jelle J. Sijtsema, Jaap J. A. Denissen and Stefan Bogaerts in Assessment

## Notes

1. Throughout the paper, measurement procedures will be described as direct and indirect, and outcomes or effects of the assessed psychological attributes as explicit and implicit (cf. de Houwer, 2006).

2. Initially, we also opted for developing an IAT for sexual entitlement. However, after several attempts to create two associated pairings of concepts representing the Sexual Entitlement IT, we concluded that creating an IAT for this complex construct, including conditions and several associations, was not possible. Efforts have been made to assess this IT via IAT, by using “Me” and “Sexual” (congruent) and “not mine” and semantically opposed but phonetically matched paired with non-sexual words (incongruent) (see Mihailides et al., 2004). However, because “a mine-sexual implicit association may reflect a more generalized construct to do with the sexual self, rather than the entitled sexual self” (Mihailides et al., 2004, p. 348), we argued that the lack of clarity of the meaning of the paired constructs is problematic and would render these concepts not usable in the present study.

3. The significant difference between participants was calculated using the reliable change formula (see Jacobson & Truax, 1992). A reliable change is typically used to calculate significant pre- and posttreatment change on one measure, based on the internal consistency and standard deviation of that measure. To accommodate the index for use with two measures, we opted for a conservative approach by using the lower bound internal consistency and the higher bound standard deviation in our calculations. However, we also computed a more liberal estimate by taking the average between the internal consistency and standard deviation between the indirect tests.

4. Based on the suggestion of the reviewer, we also ”corrected” for the attenuation of correlations due to low reliability according to the formula as described on p.137 of Murphy and Davidshofer (1988). Results can be found in the online supplementary material.

## References

[bibr1-10731911241245009] Abbey A. Parkhill M. R. Koss M. P. (2005). The effects of frame of reference on responses to questions about sexual assault victimization and perpetration. Psychology of Women Quarterly, 29(4), 364–373. 10.1111/j.1471-6402.2005.00236.x 26451071 PMC4594833

[bibr2-10731911241245009] Babchishin K. M. Nunes K. L. Hermann C. A. (2013). The validity of Implicit Association Test (IAT) measures of sexual attraction to children: A meta-analysis. Archives of Sexual Behavior, 42(3), 487–499. https://doi.org/10.1007/s10508-012-0022-8\ 23150101 10.1007/s10508-012-0022-8

[bibr3-10731911241245009] Banse R. Messer M. Fischer I. (2014). Predicting aggressive behavior with the aggressiveness-IAT. Aggressive Behavior, 41(1), 65–83. 10.1002/ab.21574 27539875

[bibr4-10731911241245009] Banse R. Schmidt A. F. Clarbour J. (2010). Indirect measures of sexual interest in child sex offenders: A multi-method approach. Criminal Justice and Behavior, 37(3), 319–335. 10.1177/0093854809357598

[bibr5-10731911241245009] Bar-Anan Y. Nosek B. A. (2014). A comparative investigation of seven indirect attitude measures. Behavior Research Methods, 46, 668–688. 10.3758/s13428-013-0410-6 24234338

[bibr6-10731911241245009] Barnes-Holmes D. Barnes-Holmes Y. Power P. Hayden E. Milne R. Stewart I. (2006). Do you really know what you believe? Developing the Implicit Relational Assessment Procedure (IRAP) as a direct measure of implicit beliefs. The Irish Psychologist, 32(7), 169–177.

[bibr7-10731911241245009] Barnes-Holmes D. Barnes-Holmes Y. Stewart I. Boles S. (2010). A sketch of the Implicit Relational Assessment Procedure (IRAP) and the Relational Elaboration and Coherence (REC) model. The Psychological Record, 60(3), 527–542. 10.1007/BF03395726

[bibr8-10731911241245009] Barnes-Holmes D. Hayden E. Barnes-Holmes Y. Stewart I. (2008). The implicit relational assessment procedure (IRAP) as a response-time and event-related-potentials methodology for testing natural verbal relations: A preliminary study. The Psychological Record, 58(4), 497–515. 10.1007/BF03395634

[bibr9-10731911241245009] Barnes-Holmes D. Murphy A. Barnes-Holmes Y. Stewart I. (2011). The Implicit Relational Assessment Procedure: Exploring the impact of private versus public contexts and the response latency criterion on pro-white and anti-black stereotyping among white Irish individuals. Psychological Record, 60(1), 57–79. 10.1007/BF03395694

[bibr10-10731911241245009] Bland J. Altman D. (1997). Statistics notes: Cronbach’s alpha. BMJ, 314, Article 572. 10.1136/bmj.314.7080.572 PMC2126061 9055718

[bibr11-10731911241245009] Blanton H. Jaccard J. Gonzales P. M. Christie C. (2006). Decoding the implicit association test: Implications for criterion prediction. Journal of Experimental Social Psychology, 42(2), 192–212. 10.1016/j.jesp.2005.07.003

[bibr12-10731911241245009] Blumenthal S. Gray N. S. Shuker R. Wood H. Fonagy P. Allonby M. Flynn A. Takala T. Snowden R. J. (2019). Implicit measurement of violence-related cognitions. Psychology of Violence, 9(2), 235–243. 10.1037/vio0000194

[bibr13-10731911241245009] Brysbaert M. (2019). How many words do we read per minute? A review and meta-analysis of reading rate. Journal of Memory and Language, 109, 104047. 10.1016/j.jml.2019.104047

[bibr14-10731911241245009] Buss A. H. Perry M. (1992). The Aggression Questionnaire. Journal of Personality and Social Psychology, 63(3), 452–459. 10.1037/0022-3514.63.3.452 1403624

[bibr15-10731911241245009] Buttrick N. Axt J. Ebersole C. R. Huband J. (2020). Re-assessing the incremental predictive validity of Implicit Association Tests. Journal of Experimental Social Psychology, 88, 103941. 10.1016/j.jesp.2019.103941

[bibr16-10731911241245009] Campbell D. T. Fiske D. W. (1959). Convergent and discriminant validation by the multitrait-multimethod matrix. Psychological Bulletin, 56(2), 81–105. 10.1037/h0046016 13634291

[bibr17-10731911241245009] Cohen J. (1988). Statistical power analysis for the behavioral sciences (2nd ed.). Lawrence Erlbaum.

[bibr18-10731911241245009] Cohen J. (1992). A power primer. Psychological Bulletin, 112(1), 155–159. 10.1037//0033-2909.112.1.155 19565683

[bibr19-10731911241245009] Cyders M. A. Coskunpinar A. (2011). Measurement of constructs using self-report and behavioral lab tasks: Is there overlap in nomothetic span and construct representation for impulsivity? Clinical Psychology Review, 31(6), 965–982. 10.1016/j.cpr.2011.06.001 21733491

[bibr20-10731911241245009] Dawson D. L. Barnes-Holmes D. Gresswell D. M. Hart A. J. Gore N. J. (2009). Assessing the implicit beliefs of sexual offenders using the implicit relational assessment procedure: A first study. Sexual Abuse: A Journal of Research and Treatment, 21(1), 57–75. 10.1177/1079063208326928 19218478

[bibr21-10731911241245009] De Houwer J . (2002). The implicit association test as a tool for studying dysfunctional associations in psychopathology: Strengths and limitations. Journal of Behavior Therapy and Experimental Psychiatry, 33(2), 115–133. 10.1016/S0005-7916(02)00024-1 12472175

[bibr22-10731911241245009] De Houwer J . (2003). The extrinsic affective Simon task. Experimental Psychology, 50(2), 77–85. 10.1026//1618-3169.50.2.77 12693192

[bibr23-10731911241245009] De Houwer J. (2006). What Are Implicit Measures and Why Are We Using Them? In Wiers R. W. Stacy A. W. (Eds.), Handbook of implicit cognition and addiction (pp. 11–28). Sage Publications

[bibr24-10731911241245009] De Houwer J. Heider N. Spruyt A. Roets A. Hughes S . (2015). The relational responding task: Towards a new implicit measure of beliefs. Frontier in Psychology, 6, Article 319. 10.3389/fpsyg.2015.00319 PMC4371587 25852624

[bibr25-10731911241245009] DeVellis R. (2003). Scale development: Theory and applications: Theory and application. Sage.

[bibr26-10731911241245009] Drake C. E. Primeaux S. Thomas J. (2018). Comparing implicit gender stereotypes between women and men with the Implicit Relational Assessment Procedure. Gender Issues, 35, 3–20. 10.1007/s12147-017-9189-6

[bibr27-10731911241245009] Edwards K. M. Gidycz C. A. Desai A. D. (2012). Men’s reactions to participating in interpersonal violence research. Journal of Interpersonal Violence, 27(18), 3683–3700. https://doi.org/10.1177%2F0886260512447576 22809816 10.1177/0886260512447576

[bibr28-10731911241245009] Faul F. Erdfelder E. Buchner A. Lang A. G. (2009). Statistical power analyses using G*Power 3.1: Tests for correlation and regression analyses. Behavior Research Methods, 41(4), 1149–1160. 10.3758/BRM.41.4.1149 19897823

[bibr29-10731911241245009] Fazio R. H. (2007). Attitudes as object-evaluation associations of varying strength. Social Cognition, 25(5), 603–637. 10.1521/soco.2007.25.5.603 19424447 PMC2677817

[bibr30-10731911241245009] Gannon T. A. Polaschek D. L. L. (2006). Cognitive distortions in child molesters: A re-examination of key theories and research. Clinical Psychology Review, 26(8), 1000–1019. 10.1016/j.cpr.2005.11.010 16480803

[bibr31-10731911241245009] Gawronski B. (2002). What does the Implicit Association Test measure? A test of the convergent and discriminant validity of prejudice-related IATs. Experimental Psychology, 49(3), 171–180. 10.1026//1618-3169.49.3.171 12152361

[bibr32-10731911241245009] Gawronski B. (2009). Ten frequently asked questions about implicit measures and their frequently supposed, but not entirely correct answers. Canadian Psychology, 50(3), 141–150. https://doi.org/10,1037/a0013848

[bibr33-10731911241245009] Gawronski B. Bodenhausen G. V. (2006). Associative and propositional processes in evaluation: An integrative review of implicit and explicit attitude change. Psychological Bulletin, 132(5), 692–731. 10.1037/0033-2909.132.5.692 16910748

[bibr34-10731911241245009] Gawronski B. Deutsch R. Banse R. (2011). Response interference tasks as indirect measures of automatic associations. In Klauer K. C. Stahl C. Voss A. (Eds.), Cognitive methods in social psychology (pp. 78–123). Guilford Press.

[bibr35-10731911241245009] Glashouwer K. A. Bennik E. C. de Jong P. J. Spruyt A. (2018). Implicit measures of actual versus ideal body image: Relations with self-reported body dissatisfaction and dieting behaviors. Cognitive Therapy and Research, 42(5), 622–635. 10.1007/s10608-018-9917-6 30237650 PMC6132988

[bibr36-10731911241245009] Greenwald A. G. Banaji M. R. (1995). Implicit social cognition: Attitudes, self-esteem, and stereotypes. Psychological Review, 102(1), 4–27. 10.1037/0033-295X.102.1.4 7878162

[bibr37-10731911241245009] Greenwald A. G. Lai C. K. (2020). Implicit social cognition. Annual Review of Psychology, 71, 419–445. 10.1146/annurev-psych-010419-050837 31640465

[bibr38-10731911241245009] Greenwald A. G. McGhee D. E. Schwartz J. L. K. (1998). Measuring individual differences in implicit cognition: The Implicit Association Test. Journal of Personality and Social Psychology, 74(6), 1465–1480. 10.1037/0022-3514.74.6.1464 9654756

[bibr39-10731911241245009] Greenwald A. G. Nosek B. A. Banaji M. R. (2003). Understanding and using the Implicit Association Test: I. An improved scoring algorithm. Journal of Personality and Social Psychology, 85(2), 197–216. 10.1037/0022-3514.85.2.197 12916565

[bibr40-10731911241245009] Gregg A. P. Seibt B. Banaji M. R. (2006). Easier done than undone: Asymmetry in the malleability of implicit preferences. Journal of Personality and Social Psychology, 90(1), 1–20. 10.1037/0022-3514.90.1.1 16448307

[bibr41-10731911241245009] Hanson R. K. Morton-Bourgon K. E. (2005). The characteristics of persistent sexual offenders: A meta-analysis of recidivism studies. Journal of Consulting and Clinical Psychology, 73(6), 1154–1163. 10.1037/0022-006X.73.6.1154 16392988

[bibr42-10731911241245009] Hayes S. C. Barnes-Holmes D. Roche B. (Eds.). (2001). Relational frame theory: A post-Skinnerian account of human language and cognition. Springer Science & Business Media. 10.1016/s0065-2407(02)80063-5 11605362

[bibr43-10731911241245009] Heider N. Spruyt A. De Houwer J. (2018). Body dissatisfaction revisited: On the importance of implicit beliefs about actual and ideal body image. Psychologica Belgica, 57(4), 158–173. 10.5334/pb.362 30479799 PMC6194529

[bibr44-10731911241245009] Helmus L. Hanson R. K. Babchinshin K. M. Mann R. E. (2013). Attitudes supportive of sexual offending predict recidivism: A meta-analysis. Trauma, Violence, & Abuse, 14(1), 34–53. 10.1177/152483801262244 23117551

[bibr45-10731911241245009] Hughes S. Barnes-Holmes D. (2013). A functional approach to the study of implicit cognition: The IRAP and the REC model. In Roche B. Dymond S. (Eds.), Advances in relational frame theory and contextual behavioral science: Research and applications (pp. 97–126). New Harbinger.

[bibr46-10731911241245009] Hughes S. Barnes-Holmes D. De Houwer J. (2011). The dominance of associative theorizing in implicit attitude research: Propositional and behavioral alternatives. The Psychological Record, 61(3), 465–496. 10.1007/BF03395772

[bibr47-10731911241245009] Hussey I. Drake C. E. (2020). The Implicit Relational Assessment Procedure demonstrates poor internal consistency and test-retest reliability: A meta-analysis (Version 7). 10.31234/osf.io/ge3k7

[bibr48-10731911241245009] Ito T. A. Friedman N. P. Bartholow B. D. Correll J. Loersch C. Altamirano L. J. Miyake A. (2015). Toward a comprehensive understanding of executive cognitive function in implicit racial bias. Journal of Personality and Social Psychology, 108(2), 187–218. 10.1037/a0038557 25603372 PMC4354845

[bibr49-10731911241245009] Jacobson N. S. Truax P. (1992). Clinical significance: A statistical approach to defining meaningful change in psychotherapy research. In Kazdin A. E. (Ed.), Methodological issues & strategies in clinical research (pp. 631–648). American Psychological Association. 10.1037/10109-042 2002127

[bibr50-10731911241245009] Kalmus E. Beech A. R. (2005). Forensic assessment of sexual interest: A review. Aggression and Violent Behavior, 10(2), 193–217. 10.1016/j.avb.2003.12.002

[bibr51-10731911241245009] Kanters T. Hornsveld R. H. J. Nunes K. L. Huijding J. Zwets A. J. Snowden R. J. Muris P. van Marle H. J. C. (2016). Are child abusers sexually attracted to submissiveness assessment of sex-related cognition with the Implicit Association Test? Sexual Abuse: A Journal of Research and Treatment, 28(5), 448–468. 10.1177/1079063214544330 25079778

[bibr52-10731911241245009] Li A. Bagger J. (2007). The Balanced Inventory of Desirable Responding (BIDR) a reliability generalization study. Educational and Psychological Measurement, 67(3), 525–544. 10.1177/0013164406292087

[bibr53-10731911241245009] Lynam D. R. Miller J. D. (2019). On the ubiquity and importance of antagonism. In Miller J. D. Lynam D. R. (Eds.), The handbook of antagonism: Conceptualizations, assessment, consequences, and treatment of the low end of agreeableness (pp. 1–24). Elsevier Academic Press. 10.1016/B978-0-12-814627-9.00001-3

[bibr54-10731911241245009] Meesters C. Muris P. Bosma H. Schouten E. Beuving S. (1996). Psychometric evaluation of the Dutch version of the Aggression Questionnaire. Behaviour Research and Therapy, 34(10), 839–843. 10.1016/0005-7967(96)00065-4 8952127

[bibr55-10731911241245009] Mihailides S. Devilly G. J. Ward T. (2004). Implicit cognitive distortions and sexual offending. Sexual Abuse: A Journal of Research and Treatment, 16(4), 333–350. 10.1177/107906320401600406 15560415

[bibr56-10731911241245009] Müller F. Rothermund K. (2019). The Propositional Evaluation Paradigm: Indirect assessment of personal beliefs and attitudes. Frontier in Psychology, 10(7), 2385. 10.3389/fpsyg.2019.02385 PMC6853888 31787908

[bibr57-10731911241245009] Murphy K. R. Davidshofer C. O. (1988). Psychological testing. Principles, and applications, Englewood Cliffs.

[bibr58-10731911241245009] Nosek B. A. (2005). Moderators of the relationship between implicit and explicit evaluation. Journal of Experimental Psychology: General, 134, 565–584. 10.1037/0096-3445.134.4.565 16316292 PMC1440676

[bibr59-10731911241245009] Nosek B. A. (2007). Implicit-explicit relations. Current Directions in Psychological Science, 16(2), 65–69. 10.1111/j.1467-8721.2007.00477.x

[bibr60-10731911241245009] Nosek B. A. Greenwald A. G. Banaji M. R. (2007). The Implicit Association Test at age 7: A methodological and conceptual review. In Bargh J. A. (Ed.), Automatic processes in social thinking and behavior (pp. 265–292). Psychology Press.

[bibr61-10731911241245009] Nosek B. A. Smyth F. L. (2007). A multitrait-multimethod validation of the implicit association test. Experimental Psychology, 54(1), 14–29. 10.1027/1618-3169.54.1.14 17341011

[bibr62-10731911241245009] Noteborn M. G. C. Hildebrand M. Sijtsema J. J. Bogaerts S. Denissen J. J. A. (2024). Validation of a Dutch short form of the Balanced Inventory of Desirable Responding (BIDR version 6): Comparing polytomous and dichotomous scoring methods in a multidimensional framework [Manuscript submitted for publication]. 10.3389/fpsyg.2025.1532969 PMC12202607 40584060

[bibr63-10731911241245009] Nunnally J. C. Bernstein I. H. (1994). Psychometric theory (3rd ed.). McGraw-Hill.

[bibr64-10731911241245009] Oswald F. L. Mitchell G. Blanton H. Jaccard J. Tetlock P. E. (2013). Predicting ethnic and racial discrimination: A meta-analysis of IAT criterion studies. Journal of Personality and Social Psychology, 105(2), 171–192. 10.1037/a0032734 23773046

[bibr65-10731911241245009] Parling T. Cernvall M. Stewart I. Barnes-Holmes D. Ghaderi A. (2012). Using the implicit relational assessment procedure to compare implicit pro-thin/anti-fat attitudes of patients with anorexia nervosa and non-clinical controls. Eating Disorders, 20(2), 127–143. 10.1080/10640266.2012.654056 22364344

[bibr66-10731911241245009] Paulhus D. L. (1984). Two-component models of socially desirable responding. Journal of Personality and Social Psychology, 46(3), 598–609. 10.1037/0022-3514.46.3.598

[bibr67-10731911241245009] Polaschek D. L. L. Gannon T. A. (2004). The implicit theories of rapists: What convicted offenders tell us. Sexual Abuse, 16(4), 299–314. 10.1177/107906320401600404 15560413

[bibr68-10731911241245009] Polaschek D. L. L. Ward T. (2002). The implicit theory of potential rapists: What our questionnaires tell us. Aggression and Violent Behavior, 7(4), 385–406. 10.1016/S1359-1789(01)00063-5

[bibr69-10731911241245009] Ranganath K. A. Smith C. T. Nosek B. A. (2008). Distinguishing automatic and controlled components of attitudes from direct and indirect measurement methods. Journal of Experimental Social Psychology, 44(2), 386–396. 10.1016/j.jesp.2006.12.008 18443648 PMC2350226

[bibr70-10731911241245009] Schmidt A. F. Banse R. Imhoff R. (2015). Indirect measures in forensic contexts. In Ortner T. M. van de Vijver F. J. R. (Eds.), Behavior-based assessment in psychology: Going beyond self-report in the personality, affective, motivation, and social domains (pp. 173–194). Hogrefe.

[bibr71-10731911241245009] Shorey R. C. Cornelius T. L. Bell K. M. (2011). Reactions to participating in dating violence research: Are our questions distressing participants? Journal of Interpersonal Violence, 26(14), 2890–2907. 10.1177/0886260510390956 21156687

[bibr72-10731911241245009] Timko C. A. England E. L. Herbert J. D. Forman E. M. (2010). The Implicit Relational Assessment Procedure as a measure of self-esteem. The Psychological Record, 60(4), 679–698. 10.1007/BF03395739

[bibr73-10731911241245009] Tremblay R. E. (2010). Developmental origins of disruptive behaviour problems: The “Original Sin” hypothesis, epigenetics and their consequences for prevention. Journal of Child Psychology and Psychiatry, 51(4), 341–367. http://doi.org/10.1111/j.1469-7610.2010.02211.x 20146751 10.1111/j.1469-7610.2010.02211.x

[bibr74-10731911241245009] Uziel L. (2010). Rethinking social desirability scales from impression management to interpersonally oriented self-control. Perspectives on Psychological Science, 5(3), 243–262. 10.1177/1745691610369465 26162157

[bibr75-10731911241245009] Vahey N. A. Barnes-Holmes D. Barnes-Holmes Y. (2009). A first test of the implicit relational assessment procedure as a measure of self-esteem: Irish prisoner groups and university students. The Psychological Record, 59(3), 371–387. 10.1007/BF03395670

[bibr76-10731911241245009] Ward T. (2000). Sexual offenders’ cognitive distortions as implicit theories. Aggression and Violent Behavior, 5(5), 491–507. 10.1016/S1359-1789(98)00036-6

[bibr77-10731911241245009] Ward T. Keenan T. (1999). Child molesters’s implicit theories. Journal of Interpersonal Violence, 14(8), 821–838. 10.1177/088626099014008003

[bibr78-10731911241245009] Widman L. McNulty J. K. (2010). Sexual narcissism and the perpetration of sexual aggression. Archives of Sexual Behavior, 39(4), 926–939. 10.1007/s10508-008-9461-7 19130204 PMC4112751

[bibr79-10731911241245009] Wilson T. D. (2009). Know thyself. Perspectives on Psychological Science, 4(4), 384–389. 10.1111/j.1745-6924.2009.01143.x 26158986

[bibr80-10731911241245009] Wilson T. D. Lindsey S. Schooler T. Y. (2000). A model of dual attitudes. Psychological Review, 107(1), 101–126. 10.1037/0033-295X.107.1.101 10687404

[bibr81-10731911241245009] Yeater E. Miller G. Rinehart J. Nason E. (2012). Trauma and sex surveys meet minimal risk standards: Implications for institutional review boards. Psychological Science, 23(7), 780–787. 10.1177/0956797611435131 22623507

[bibr82-10731911241245009] Young S. Cocallis K. M. (2019). Attention Deficit Hyperactivity Disorder (ADHD) in the prison system. Current Psychiatry Reports, 21, 1–9. 10.1007/s11920-019-1022-3 31037396

