# Supplementary material for: Assessing Implicit Theories in Sexual Offending Using Indirect Measures: Feasibility, Reliability, and Incremental Validity
Source: Assessment. 2024 May 7;32(3):447–69. doi: 10.1177/10731911241245009 (PMC11915770; doi:10.1177/10731911241245009)
Supplement: sj-docx-1-asm-10.1177_10731911241245009 – Supplemental material for Assessing Implicit Theories in Sexual Offending Using Indirect Measures: Feasibility, Reliability, and Incremental Validity [file sj-docx-1-asm-10.1177_10731911241245009.docx]

| Online supplementary material | | | | | | | | |  |
| --- | --- | --- | --- | --- | --- | --- | --- | --- | --- |
|  |  |  |  |  |  |  |  |  |  |
|  |  |  |  |  |  |  |  |  |  |
|  |  |  |  |  |  |  |  |  |  |
|  |  |  |  |  |  |  |  |  |  |
|  | | | | | | | | |  |
|  |  |  |  |  |  |  |  |  |  |
|  |  |  |  |  |  |  |  |  |  |
|  |  |  |  |  |  |  |  |  |  |
|  |  |  |  |  |  |  |  |  |  |
|  |  |  |  |  |  |  |  |  |  |
|  |  |  |  |  |  |  |  |  |  |
|  |  |  |  |  |  |  |  |  |  |
|  |  |  |  |  |  |  |  |  |  |
|  |  |  |  |  |  |  |  |  |  |
|  |  |  |  |  |  |  |  |  |  |
|  |  |  |  |  |  |  |  |  |  |

| Online supplementary material S1 | | | | | | | | | | | |  |
| --- | --- | --- | --- | --- | --- | --- | --- | --- | --- | --- | --- | --- |
|  |  |  |  |  |  |  |  |  |  |  |  |  |
|  |  |  |  |  |  |  |  |  |  |  |  |  |
|  |  |  |  |  |  |  |  |  |  |  |  |  |
|  |  |  |  |  |  |  |  |  |  |  |  |  |
| Online supplementary material directly referred to in the manuscript | | | | | | | | | | | |  |
|  | | | | | | | | | | | |  |
| Table S1.1 | Differences in age and educational level between participants who passes and did not pass the Implicit Relational Assessment Procedure (IRAP) criteria | | | | | | | | | | |  |
| Table S1.2 | Differences in age and educational level between participants who passed the IRAP and the RRT or IAT, or only passed RRT or IAT | | | | | | | | | | |  |
| Table S1.3 | Passing criteria and medain latencies per educational level for Aggression and Sexual entitlement. | | | | | | | | | | |  |
| Table S1.4 | Multitrait-multimethod matrix direct and indirect measures of Aggression and Sexual entitlement and additional correlations between study variables. | | | | | | | | | | |  |
| Table S1.5 | Multitrait-multimethod matrix direct and indirect measures of aggression and sexual entitlement corrected for the attenuation of correlations. | | | | | | | | | | |  |
|  | G*power power protocol current study | | | | | | | | | | |  |
|  | G*power power protocol futures studies | | | | | | | | | | |  |
|  |  |  |  |  |  |  |  |  |  |  |  |  |
|  |  |  |  |  |  |  |  |  |  |  |  |  |
|  |  |  |  |  |  |  |  |  |  |  |  |  |

| Table S1.1 | |  |  |  |  |  |  |  |  |  |
| --- | --- | --- | --- | --- | --- | --- | --- | --- | --- | --- |
| *Differences in age and educational level between participants who passes and did not pass the Implicit Relational Assessment Procedure (IRAP) criteria* | | | | | | | | | | |
|  |  |  | Aggression | | |  |  | Sexual Entitlement | | |
|  |  |  | Passed | Did not pass |  |  |  | Passed | Did not pass |  |
| Age |  |  | *Md* | *Md* | difference (*U*) |  |  | *M*(*SD*) | *M*(*SD*) | difference (*t*) |
|  | n |  | 90 | 18 |  |  | n | 80 | 28 |  |
|  |  |  | 25.00 | 24.50 | *U*= 757, *z*= -0.44 |  |  | 31.08 (14.64) | 36.86 (17.95) | t(40.283)= -1.535 |
|  |  |  |  |  |  |  |  |  |  |  |
|  |  |  | *%* | *%* | χ^2^ |  |  | *%* | *%* | χ^2^ |
|  | n |  | 91 | 17 |  |  | n | 81 | 27 |  |
| Education | |  |  |  | 4.837^a^ |  |  |  |  | 5.948^a^ |
|  | High School |  | 35.2% | 17.6% |  |  |  | 37.0% | 18.5% |  |
|  | LVE |  | 22.0% | 47.1% |  |  |  | 21.0% | 40.7% |  |
|  | HVE |  | 26.4% | 17.6% |  |  |  | 23.5% | 29.6% |  |
|  | University |  | 16.5% | 17.6% |  |  |  | 18.5% | 11.1% |  |
| *_Note._* _LVE = Low Vocational Education; HVE = High Vocational Education._ _One participant did not indicate age and is therefore not included in the analyses. Only one participant indicated elementary school as highest education and therefore not included in the analyses._  ^a^ _due to cells with expected count below 5% Fisher exact test was used. * ≤_ *_p_* _= .05, ** ≤_ *_p_* _= .001_ | | | | | | | | | | |

| Table S1.2 | |  |  |  |  |  |  |  |  |
| --- | --- | --- | --- | --- | --- | --- | --- | --- | --- |
| *Differences in age and educational level between participants who passed the IRAP and the RRT or IAT, or only passed RRT or IAT* | | | | | | | |  |  |
|  |  |  | Passed the IRAP & other measure |  | Passed only other measure |  |  |  |  |
|  |  |  | Agression | | | | |  |  |
|  |  |  | *Md* |  | *Md* |  | difference (*U*) |  |  |
| Age | RRT (n) |  | 90 |  | 15 |  |  |  |  |
|  |  |  | 25.00 |  | 25.00 |  | *U*= 592, *z*= -0.76 |  |  |
|  | IAT (n) |  | 90 |  | 16 |  |  |  |  |
|  |  |  | 25.00 |  | 25.00 |  | *U*= 636, *z*= -0.74 |  |  |
|  |  |  |  |  |  |  |  |  |  |
|  |  |  |  |  |  |  |  |  |  |
|  |  |  |  |  |  |  |  |  |  |
|  |  |  | *%* |  | *%* |  | χ^2^ |  |  |
|  | RRT (n) |  | 91 |  | 14 |  |  |  |  |
| Education | |  |  |  |  |  | 2.065^a^ |  |  |
| High School | |  | 35.2% |  | 21.4% |  |  |  |  |
| LVE | |  | 22.0% |  | 35.7% |  |  |  |  |
| HVE | |  | 26.4% |  | 21.4% |  |  |  |  |
| University | |  | 16.5% |  | 21.4% |  |  |  |  |
|  | IAT (n) |  | 91 |  | 15 |  |  |  |  |
|  |  |  |  |  |  |  | 2.863^a^ |  |  |
| High School | |  | 35.2% |  | 20.0% |  |  |  |  |
| LVE | |  | 22.0% |  | 40.0% |  |  |  |  |
| HVE | |  | 26.4% |  | 20.0% |  |  |  |  |
| University | |  | 16.5% |  | 20.0% |  |  |  |  |
|  |  | Sexual Entitlement | | | | | | | |
|  |  |  | *M*(*SD*) |  | *M*(*SD*) |  | difference (*t*) |  |  |
| Age | RRT (n) |  | 80 |  | 24 |  |  |  |  |
|  |  |  | 31.14 (14.722) |  | 34.54 (16.15) |  | t(101)= -0.969 |  |  |
|  |  |  |  |  |  |  |  |  |  |
|  |  |  |  |  |  |  |  |  |  |
|  |  |  |  |  |  |  |  |  |  |
|  |  |  | *%* |  | *%* |  | χ^2^ |  |  |
|  | RRT (n) |  | 80 |  | 23 |  |  |  |  |
| Education | |  |  |  |  |  | 2.939^a^ |  |  |
| High School | |  | 36.3% |  | 21.7% |  |  |  |  |
| LVE | |  | 21.3% |  | 30.4% |  |  |  |  |
| HVE | |  | 23.8% |  | 34.8% |  |  |  |  |
| University | |  | 18.8% |  | 13.0% |  |  |  |  |
| *_Note._*_LVE = Low vocational education, HVE = High vocational education. One participant did not indicate age and is therefore not included in the analyses. Only one participant indicated elementary school as highest education and therefore not included in the analyses.._ ^a^ _due to cells with expected count below 5% Fisher exact test was used. * ≤_ *_p_* _= .05, ** ≤_ *_p_* _= .001_ | | | | | | | |  |  |

| Table S1.3 | |  |  |  |  |  |  |  |  |  |  |  |  |  |  |  |  |  |
| --- | --- | --- | --- | --- | --- | --- | --- | --- | --- | --- | --- | --- | --- | --- | --- | --- | --- | --- |
| *Passing criteria and medain latencies per educational level for Aggression and Sexual entitlement.* | | | | | | | | | | | | | | | |  |  |  |
|  |  | Implicit Relational Assessment Procedure (IRAP) | | | | |  | Relational Responding Task | | | | |  | Implicit Association Test | |  |  |  |
|  |  | Agression | |  | Sexual entitlement | |  | Aggression | |  | Sexual entitlement | |  | Aggression | |  |  |  |
|  |  |  | |  |  | |  |  |  |  |  |  |  |  |  |  |  |  |
|  | Total *n* | 109 | |  | 109 | |  | 109 | |  | 108 | |  | 109 | |  |  |  |
| Passed the criteria (*n*) | | 91 | |  | 81 | |  | 106 | |  | 104 | |  | 107 | |  |  |  |
|  |  |  |  |  |  |  |  |  |  |  |  |  |  |  |  |  |  |  |
| Education | | *Md* | *(n)* |  | *Md* | *(n)* |  | *Md* | *(n)* |  | *Md* | *(n)* |  | *Md* | *(n)* |  |  |  |
| High School | | 1332.30 | (32) |  | 1867.81 | (30) |  | 1076.59 | (35) |  | 1715.60 | (34) |  | 841.13 | (35) |  |  |  |
| Low vocational education | | 1709.21 | (20) |  | 2319.50 | (17) |  | 1409.91 | (25) |  | 2182.13 | (24) |  | 958.13 | (26) |  |  |  |
| High vocational education | | 1634.47 | (24) |  | 2187.62 | (19) |  | 1531.90 | (27) |  | 2140.06 | (27) |  | 1026.50 | (27) |  |  |  |
| University | | 1528.13 | (15) |  | 2056.49 | (15) |  | 1326.96 | (18) |  | 1810.61 | (18) |  | 916.82 | (18) |  |  |  |
| *_Note._* _One participant did not finish the RRT Entitlement due to time constrains, therefore the total number of participants is 108 instead of 109._ | | | | | | | | | | | | | | | |  |  |  |
|  |  |  |  |  |  |  |  |  |  |  |  |  |  |  |  |  |  |  |
|  |  |  |  |  |  |  |  |  |  |  |  |  |  |  |  |  |  |  |

| Table S1.4. | | |  | | |  | |  | |  | |  | |  | |  | |  | |  | |  | |  |  | |  | | |  |  | |  | |  | |  | | |  |  |  |  |  |  |  |  |  |  |
| --- | --- | --- | --- | --- | --- | --- | --- | --- | --- | --- | --- | --- | --- | --- | --- | --- | --- | --- | --- | --- | --- | --- | --- | --- | --- | --- | --- | --- | --- | --- | --- | --- | --- | --- | --- | --- | --- | --- | --- | --- | --- | --- | --- | --- | --- | --- | --- | --- | --- |
| *Multitrait-multimethod matrix direct and indirect measures of Aggression and Sexual entitlement and additional correlations between study variables.* | | | | | | | | | | | | | | | | | | | | | | | | | | | | | | | | | | | | |  | | |  |  |  |  |  |  |  |  |  |  |
|  |  | |  | | |  | | Indirect measures | | | | | | | | | | | | | |  | |  | Direct measures | | | | |  |  | |  | |  | |  | | |  |  |  |  |  |  |  |  |  |  |
|  |  | |  | | |  | | IRAP | | | |  | | RRT | | | |  | | IAT | | | |  | self-report | | | | |  |  | |  | |  | |  | | |  |  |  |  |  |  |  |  |  |  |
|  |  | |  | | |  | | 1 | | 2 | |  | | 1 | | 2 | |  | | 1 | | 2 | |  | 3^a^ | | 4 | | |  | 5 | | 6 | | 7 | | 8 | | | 9 |  |  |  |  |  |  |  |  |  |
| IRAP | 1 | | *D*score Aggression | | | *r* | | (.28) | |  | |  | |  | |  | |  | |  | |  | |  |  | |  | | |  |  | |  | |  | |  | | |  |  |  |  |  |  |  |  |  |  |
|  |  |  |  |  |  | *n* | | 91 | |  | |  | |  | |  | |  | |  | |  | |  |  | |  | | |  |  | |  | |  | |  | | |  |  |  |  |  |  |  |  |  |  |
|  | 2 | | *D*score Sexual entitlement | | | *r* | | .32** | | (.58) | |  | |  | |  | |  | |  | |  | |  |  | |  | | |  |  | |  | |  | |  | | |  |  |  |  |  |  |  |  |  |  |
|  |  |  |  |  |  | *n* | | 76 | | 81 | |  | |  | |  | |  | |  | |  | |  |  | |  | | |  |  | |  | |  | |  | | |  |  |  |  |  |  |  |  |  |  |
| RRT | 1 | | *D*score Aggression | | | *r* | | .23* | | -.01 | |  | | (.54) | |  | |  | |  | |  | |  |  | |  | | |  |  | |  | |  | |  | | |  |  |  |  |  |  |  |  |  |  |
|  |  |  |  |  |  | *n* | | 91 | | 76 | |  | | 91 | |  | |  | |  | |  | |  |  | |  | | |  |  | |  | |  | |  | | |  |  |  |  |  |  |  |  |  |  |
|  | 2 | | *D*score Sexual entitlement | | | *r* | | .15 | | .35** | |  | | .01 | | (.61) | |  | |  | |  | |  |  | |  | | |  |  | |  | |  | |  | | |  |  |  |  |  |  |  |  |  |  |
|  |  |  |  |  |  | *n* | | 76 | | 80 | |  | | 76 | | 80 | |  | |  | |  | |  |  | |  | | |  |  | |  | |  | |  | | |  |  |  |  |  |  |  |  |  |  |
| IAT | 1 | | *D*score Aggression | | | *r* | | .18 | | -.01 | |  | | .05 | | .06 | |  | | (.66) | |  | |  |  | |  | | |  |  | |  | |  | |  | | |  |  |  |  |  |  |  |  |  |  |
|  |  |  |  |  |  | *n* | | 90 | | 76 | |  | | 91 | | 76 | |  | | 91 | |  | |  |  | |  | | |  |  | |  | |  | |  | | |  |  |  |  |  |  |  |  |  |  |
|  | 2 | | *D*score Sexual entitlement | | | *r* | | n.a. | | n.a. | |  | | n.a. | | n.a. | |  | | n.a. | | n.a. | |  |  | |  | | |  |  | |  | |  | |  | | |  |  |  |  |  |  |  |  |  |  |
|  |  |  |  |  |  | *n* | |  | |  | |  | |  | |  | |  | |  | |  | |  |  | |  | | |  |  | |  | |  | |  | | |  |  |  |  |  |  |  |  |  |  |
| self-report | 3 | | Aggression^a^ | | | *r* | | .22* | | .01 | |  | | .15 | | -.06 | |  | | .09 | | n.a. | |  | (.83) | |  | | |  |  | |  | |  | |  | | |  |  |  |  |  |  |  |  |  |  |
|  |  |  |  |  |  | *n* | | 91 | | 80 | |  | | 91 | | 80 | |  | | 91 | |  | |  | 91 | |  | | |  |  | |  | |  | |  | | |  |  |  |  |  |  |  |  |  |  |
|  | 4 | | Sexual entitlement | | | *r* | | .07 | | .19 | |  | | -.02 | | .11 | |  | | -.10 | | n.a. | |  | -.09 | | (.62) | | |  |  | |  | |  | |  | | |  |  |  |  |  |  |  |  |  |  |
|  |  |  |  |  |  | *n* | | 91 | | 80 | |  | | 91 | | 80 | |  | | 91 | |  | |  | 91 | | 91 | | |  |  | |  | |  | |  | | |  |  |  |  |  |  |  |  |  |  |
|  |  | |  | | |  | |  | |  | |  | |  | |  | |  | |  | |  | |  |  | |  | | |  |  | |  | |  | |  | | |  |  |  |  |  |  |  |  |  |  |
|  | 5 | | Informant reported aggression | | | *r* | | .03 | | -.11 | |  | | .22* | | -.29* | |  | | .26* | | n.a. | |  | .59** | | -.20 | | |  |  | |  | |  | |  | | |  |  |  |  |  |  |  |  |  |  |
|  |  |  |  |  |  | *n* | | 84 | | 77 | |  | | 84 | | 77 | |  | | 84 | |  | |  | 84 | | 84 | | |  |  | |  | |  | |  | | |  |  |  |  |  |  |  |  |  |  |
|  | 6 | | Sexual aggressive behavior | | | *r* | | .07 | | .16 | |  | | .08 | | -.05 | |  | | -.06 | | n.a. | |  | .17 | | .33** | | |  | .02 | |  | |  | |  | | |  |  |  |  |  |  |  |  |  |  |
|  |  |  |  |  |  | *n* | | 91 | | 80 | |  | | 91 | | 80 | |  | | 91 | |  | |  | 91 | | 91 | | |  | 84 | |  | |  | |  | | |  |  |  |  |  |  |  |  |  |  |
|  | 7 | | IM | | | *r* | | -.11 | | -.03 | |  | | -.23* | | -.11 | |  | | -.17 | | n.a. | |  | -.30** | | -.27* | | |  | -.15 | | -.30* | |  | |  | | |  |  |  |  |  |  |  |  |  |  |
|  |  |  |  |  |  | *n* | | 91 | | 80 | |  | | 91 | | 80 | |  | | 91 | |  | |  | 91 | | 91 | | |  | 84 | | 91 | |  | |  | | |  |  |  |  |  |  |  |  |  |  |
|  | 8 | | SDE | | | *r* | | -.08 | | .04 | |  | | -.22* | | -.09 | |  | | .05 | | n.a. | |  | -.10 | | .12 | | |  | -.09 | | -.16 | | .39** | |  | | |  |  |  |  |  |  |  |  |  |  |
|  |  |  |  |  |  | *n* | | 91 | | 80 | |  | | 91 | | 80 | |  | | 91 | |  | |  | 91 | | 91 | | |  | 84 | | 91 | | 91 | |  | | |  |  |  |  |  |  |  |  |  |  |
|  | 9 | | Age | | | *r* | | .03 | | .17 | |  | | -.37** | | .09 | |  | | -.13 | | n.a. | |  | .12 | | -.04 | | |  | -.05 | | .17 | | .07 | | .00 | | |  |  |  |  |  |  |  |  |  |  |
|  |  |  |  |  |  | *n* | | 90 | | 79 | |  | | 90 | | 79 | |  | | 90 | |  | |  | 90 | | 90 | | |  | 83 | | 90 | | 90 | | 90 | | |  |  |  |  |  |  |  |  |  |  |
| *_Notes._* _MTMM can be found in the upper part of the table. Monotrait-heteromethod correlations (validity diagonals) are presented in gray. Reliability diagonals are the values in parentheses. Internal consistency for the indirect measures is calculated using Split half reliability with spearman brown correction. Internal consistency for the other measures is calculated using cronbach's alpha. Each heterotrait-monomethod correlation is enclosed by a solid line. Each heterotrait-heteromethod correlation is enclosed by a broken line. Monomethodblocks consist out of the Reliability diagonals and the heterotrait-monomethod correlations. Heteromethodblocks consist of Monotrait-heteromethod correlations and heterotrait-heteromethod correlations. Sexual aggressive behavior is a dummy variable with having shown sexually aggressive behavior serving as the reference group. A Due to non-normal distribution Spearman Rho was calculated. **Correlation is significant at the .001 level (2-tailed). *Correlation is significant at the .05 level (2-tailed). For a more clear visual overview of the MTMM analyses, the variable Sexual entitlement IAT was included in the table_. | | | | | | | | | | | | | | | | | | | | | | | | | | | | | | | | | | | | | | | | |  |  |  |  |  |  |  |  |  |
|  |  |  |  |  |  |  |  |  |  |  |  |  |  |  |  |  |  |  |  |  |  |  |  |  |  |  |  |  |  |  |  |  |  |  |  |  |  |  |  |  |  |  |  |  |  |  |  |  |  |
|  |  | |  | | |  | |  | |  | |  | |  | |  | |  | |  | |  | |  |  | |  | | |  |  | |  | |  | |  | | |  |  |  |  |  |  |  |  |  |  |
| Table S1.5 | | | |  |  | |  | |  | |  | |  | |  | |  | |  | |  | |  | | |  | |  |  | | |  | |  | |  | |  |  |  |  |  |  |  |  |  |  |  |  |
| *Multitrait-multimethod matrix direct and indirect measures of aggression and sexual entitlement corrected for the attenuation of correlations.* | | | | | | | | | | | | | | | | | | | | | | | | | | | | | | | | | | | | | |  |  |  |  |  |  |  |  |  |  |  |  |
|  | |  | |  | Indirect measures | | | | | | | | | | | | | |  | |  | | Direct measures | | | | |  |  | | |  | |  | |  | |  |  |  |  |  |  |  |  |  |  |  |  |
|  | |  | |  | IRAP | | | |  | | RRT | | | |  | | IAT | | | |  | | self-report | | | | |  |  | | |  | |  | |  | |  |  |  |  |  |  |  |  |  |  |  |  |
|  | |  | |  | 1 | | 2 | |  | | 1 | | 2 | |  | | 1 | | 2 | |  | | 3^a^ | | | 4 | |  | 5 | | | 6 | | 7 | | 8 | |  |  |  |  |  |  |  |  |  |  |  |  |
| IRAP | | 1. | | *D*score Aggression | (.28) | |  | |  | |  | |  | |  | |  | |  | |  | |  | | |  | |  |  | | |  | |  | |  | |  |  |  |  |  |  |  |  |  |  |  |  |
|  |  | 2. | | *D*score Sexual entitlement | .79** | | (.58) | |  | |  | |  | |  | |  | |  | |  | |  | | |  | |  |  | | |  | |  | |  | |  |  |  |  |  |  |  |  |  |  |  |  |
| RRT | | 1. | | *D*score Aggression | .59** | | -.01 | |  | | (.54) | |  | |  | |  | |  | |  | |  | | |  | |  |  | | |  | |  | |  | |  |  |  |  |  |  |  |  |  |  |  |  |
|  |  | 2. | | *D*score Sexual entitlement | .36** | | .59** | |  | | .02 | | (.61) | |  | |  | |  | |  | |  | | |  | |  |  | | |  | |  | |  | |  |  |  |  |  |  |  |  |  |  |  |  |
| IAT | | 1. | | *D*score Aggression | .41** | | -.02 | |  | | .08 | | .10 | |  | | (.66) | |  | |  | |  | | |  | |  |  | | |  | |  | |  | |  |  |  |  |  |  |  |  |  |  |  |  |
|  |  | 2. | | *D*score Sexual entitlement | n.a. | | n.a. | |  | | n.a. | | n.a. | |  | | n.a. | | n.a. | |  | |  | | |  | |  |  | | |  | |  | |  | |  |  |  |  |  |  |  |  |  |  |  |  |
| self-report | | 3. | | Aggression^a^ | .46** | | .01 | |  | | 0.23* | | -.08 | |  | | .12 | | n.a. | |  | | (.83) | | |  | |  |  | | |  | |  | |  | |  |  |  |  |  |  |  |  |  |  |  |  |
|  |  | 4. | | Sexual entitlement | .16 | | .32** | |  | | -.04 | | .18 | |  | | -.15 | | n.a. | |  | | -.13 | | | (.62) | |  |  | | |  | |  | |  | |  |  |  |  |  |  |  |  |  |  |  |  |
|  | |  | |  |  | |  | |  | |  | |  | |  | |  | |  | |  | |  | | |  | |  |  | | |  | |  | |  | |  |  |  |  |  |  |  |  |  |  |  |  |
|  | | 5. | | Informant reported aggression | .03 | | -.11 | |  | | .22* | | -.29* | |  | | .26* | | n.a. | |  | | .59** | | | -.20 | |  |  | | |  | |  | |  | |  |  |  |  |  |  |  |  |  |  |  |  |
|  |  | 6. | | Sexual aggressive behavior | .07 | | .16 | |  | | .08 | | -.05 | |  | | -.06 | | n.a. | |  | | .17 | | | .33** | |  | .02 | | |  | |  | |  | |  |  |  |  |  |  |  |  |  |  |  |  |
| *Note*. MTMM can be found in the upper part of the table. Monotrait-heteromethod correlations (validity diagonals) are presented in gray. Reliability diagonals are the values in parantheses. Internal consistency for the indirect measures is calculated using Split half relaibility with spearman brown correction. Internal consistency for the other meausres is calculated using cronbach's alpha. Each heterotrait-monomethod correlation is enclosed by a solid line. Each heterotrait-heteromethod correlation is enclosed by a broken line. Monomethodblocks consist out of the Reliability diagonals and the heterotrait-monomethod correlations. Heteromethodblocks consist of Monotrait-heteromethod correlations and heterotrait-heteromethod correlations. Sexual aggressive behavior is a dummy variable with having shown sexually agressive behavior serving as the reference group. ^A^ Due to non-normal distribution Spearman Rho was calculated. **Correlation is significant at the .001 level (2-tailed). *Correlation is significant at the .05 level (2-tailed). *N* differed per analyses. For Aggression *n* = 91; For Entitlement *n* = 80; n for both Entitlement and Aggression combined was 76. For correlations between self-reported measures higherst sample size was chosen (i.e. 91). For exact sample sizes used see the appendix. For a more clear visual overview of the MTMM analyses, the variable Sexual entitlement IAT was included in the table. | | | | | | | | | | | | | | | | | | | | | | | | | | | | | | | | | | | | | |  |  |  |  |  |  |  |  |  |  |  |  |
|  |  |  |  |  |  |  |  |  |  |  |  |  |  |  |  |  |  |  |  |  |  |  |  |  |  |  |  |  |  |  |  |  |  |  |  |  |  |  |  |  |  |  |  |  |  |  |  |  |  |
|  |  |  |  |  |  |  |  |  |  |  |  |  |  |  |  |  |  |  |  |  |  |  |  |  |  |  |  |  |  |  |  |  |  |  |  |  |  |  |  |  |  |  |  |  |  |  |  |  |  |

| G*power power protocol current study | |  |  |
| --- | --- | --- | --- |
|  |  |  |  |
| **F tests -** Linear multiple regression: Fixed model, R² deviation from zero | | | |
| **Analysis:** | Post hoc: Compute achieved power | |  |
| **Input:** | Effect size f² | = | 0.15 |
|  | α err prob | = | 0.05 |
|  | Total sample size | = | 80 |
|  | Number of predictors | = | 3 |
| **Output:** | Noncentrality parameter λ | = | 120,000,000 |
|  | Critical F | = | 27,249,439 |
|  | Numerator df | = | 3 |
|  | Denominator df | = | 76 |
|  | Power (1-β err prob) | = | 0.8190542 |

| G*power power protocol future studies | | |  |
| --- | --- | --- | --- |
|  |  |  |  |
| **F tests -** Linear multiple regression: Fixed model, R² deviation from zero | | | |
| **Analysis:** | A priori: Compute required sample size | |  |
| **Input:** | Effect size f² | = | 0.01 |
|  | α err prob | = | 0.05 |
|  | Power (1-β err prob) | = | 0.8 |
|  | Number of predictors | = | 3 |
| **Output:** | Noncentrality parameter λ | = | 109,500,000 |
|  | Critical F | = | 26,130,603 |
|  | Numerator df | = | 3 |
|  | Denominator df | = | 1091 |
|  | Total sample size | = | 1095 |
|  | Actual power | = | 0.8003321 |

| Online supplementary material S2 | | | | | | | | | | | |  |
| --- | --- | --- | --- | --- | --- | --- | --- | --- | --- | --- | --- | --- |
|  |  |  |  |  |  |  |  |  |  |  |  |  |
|  |  |  |  |  |  |  |  |  |  |  |  |  |
|  |  |  |  |  |  |  |  |  |  |  |  |  |
|  |  |  |  |  |  |  |  |  |  |  |  |  |
| Results 2,000, 3,000 and 5,000ms | | | | | | | | | | | |  |
|  | | | | | | | | | | | |  |
| Table S2.1 | Passing criteria for all indirect measures of aggression and sexual entitlement. | | | | | | | | | | |  |
| Table S2.2 | Mean latencies and error rates and the effects of age and education for all indirect measures of aggression and sexual entitlement. | | | | | | | | | | |  |
| Table S2.3 | Differences in age and educational level between participants who passes and did not pass the Implicit Relational Assessment Procedure (IRAP) criteria all ms | | | | | | | | | | |  |
| Table S2.4 | Differences in age and educational level between participants who passes and did not pass the Implicit Relational Assessment Procedure (IRAP) criteria all ms | | | | | | | | | | |  |
| Table S2.5 | Differences in age and educational level between participants who passed the IRAP and the RRT or IAT, or only passed RRT or IAT all ms | | | | | | | | | | |  |
| Table S2.6 | Differences in age and educational level between the different passing latencies of the IRAP all ms | | | | | | | | | | |  |
| Table S2.7 | Passing criteria and medain latencies per educational level for Aggression and Sexual entitlement all ms | | | | | | | | | | |  |
|  |  |  |  |  |  |  |  |  |  |  |  |  |
|  |  |  |  |  |  |  |  |  |  |  |  |  |
|  |  |  |  |  |  |  |  |  |  |  |  |  |

| Table S2.1 |  |  |  |  |  |  |  |  |  |  |  |  |  |  |  |  |  |  |  |
| --- | --- | --- | --- | --- | --- | --- | --- | --- | --- | --- | --- | --- | --- | --- | --- | --- | --- | --- | --- |
| *Passing criteria for all indirect measures of aggression and sexual entitlement.* | | | | | | | |  |  |  |  |  |  |  |  |  |  |  |  |
|  |  | Implicit Relational Assessment Procedure | | | | | | |  | Relational Responding Task | | | | |  | Implicit Association Test | | |  |
|  |  | Aggression | | |  | Sexual entitlement | | |  | Aggression | |  | Sexual entitlement | |  | Aggression | | |  |
|  |  | 2.068 ms + 80% correct | 3.068 ms + 80% correct | 5.068 ms + 80% correct |  | 2.291 ms + 80% correct | 3.291 ms + 80% correct | 5.291 ms + 80% correct |  |  |  |  |  |  |  |  |  |  |  |
| *N* |  | 109 | 109 | 109 |  | 109 | 109 | 109 |  | 109 | |  | 108 | |  | 109 | | |  |
| Participants deleted based on specific passing criteria IRAP | | 35 | 16 | 12 |  | 68 | 25 | 12 |  | - | |  | - | |  | - | | |  |
| *n* | | 74 | 93 | 97 |  | 41 | 84 | 97 |  | 109 | |  | 108 | |  | 109 | | |  |
|  |  |  |  |  |  |  |  |  |  |  |  |  |  |  |  |  |  |  |  |
| Participants deleted based on 2.5 std. > mean error rate | | 2 | 2 | 2 |  | 1 | 3 | 5 |  | 3 | |  | 4 | |  | 2 | | |  |
| Participants deleted based on 10% latencies < 300 ms | | 0 | 0 | 0 |  | 0 | 0 | 0 |  | 0 | |  | 0 | |  | 0 | | |  |
|  |  |  |  |  |  |  |  |  |  |  |  |  |  |  |  |  |  |  |  |
| Participants that passed the criteria (n) | | 72 | 91 | 95 |  | 40 | 81 | 92 |  | 106 | |  | 104 | |  | 107 | | |  |
|  |  |  |  |  |  |  |  |  |  |  |  |  |  |  |  |  |  |  |  |
| Participants trails latencies > 10.000 ms | | 2 | 4 | 7 |  | 0 | 3 | 10 |  | 1 | |  | 10 | |  | 2 | | |  |
| *Note.* One participant did not finish the RRT Entitlement due to time constrains, therefore the total number of participants is 108 instead of 109. | | | | | | | | | | | | | | | | | | |  |

| Table S2.5 (Continued). | |  |  |  |  |  |
| --- | --- | --- | --- | --- | --- | --- |
|  |  |  |  |  |  |  |
|  |  | Implicit Relational Assessment Procedure | | | |  |
|  |  | Aggression | | | |  |
|  |  | 5.000 ms + 80% Correct | | | |  |
|  |  | Passed the IRAP & other measure |  | Passed only other measure |  |  |
|  |  | *Md* |  | *Md* | difference (*U*) |  |
| Age | RRT (n) | 94 |  | 11 |  |  |
|  |  | 25.00 |  | 24.00 | *U*= 508, *z*= -0.09 |  |
|  | IAT (n) | 94 |  | 12 |  |  |
|  |  | 25.00 |  | 23.50 | *U*= 555, *z*= -0.09 |  |
|  |  |  |  |  |  |  |
|  |  | *%* |  | *%* | χ^2^ |  |
|  | RRT (n) | 95 |  | 10 |  |  |
| Education | |  |  |  | 2.070^a^ |  |
| High School | | 34.7% |  | 20.0% |  |  |
| LVE | | 22.1% |  | 40.0% |  |  |
| HVE | | 26.3% |  | 20.0% |  |  |
| University | | 16.8% |  | 20.0% |  |  |
|  | IAT (n) | 95 |  | 11 |  |  |
|  |  |  |  |  | 3.045^a^ |  |
| High School | | 34.7% |  | 18.2% |  |  |
| LVE | | 22.1% |  | 45.5% |  |  |
| HVE | | 26.3% |  | 18.2% |  |  |
| University | | 16.8% |  | 18.2% |  |  |
|  |  | Sexual entitlement | | | |  |
|  |  | *Md* |  | *Md* | difference (*U*) |  |
| Age | RRT (n) | 91 |  | 14 |  |  |
|  |  | 25 |  | 23 | *U*= 373, *z*= -2.54^**^, *r* =.24 |  |
|  |  | *%* |  | *%* | χ^2^ |  |
|  | RRT (n) | 91 |  | 13 |  |  |
| Education | |  |  |  | 2.588^a^ |  |
| High School | | 31.9% |  | 38.5% |  |  |
| LVE | | 22.0% |  | 38.5% |  |  |
| HVE | | 27.5% |  | 15.4% |  |  |
| University | | 18.7% |  | 7.7% |  |  |
| *_Note_*_. LVE = Low vocational education, HVE = High vocational education. One participant did not indicate age and is therefore not included in the analyses. Only one participant indicated elementary school as highest education and therefore not included in the analyses. Though chi-square analyses indicated sign differences, post-hoc analyses did not always indicate significance after applying bonferonni correction._ ^a^ _due to cells with expected count below 5% Fisher exact test was used. * ≤_ *_p_* _= .05, ** ≤_ *_p_* _= .001_ | | | | | |  |
|  |  |  |  |  |  |  |

| Online supplementary material S3 | | | | | | | | | | | |  |
| --- | --- | --- | --- | --- | --- | --- | --- | --- | --- | --- | --- | --- |
|  |  |  |  |  |  |  |  |  |  |  |  |  |
|  |  |  |  |  |  |  |  |  |  |  |  |  |
|  |  |  |  |  |  |  |  |  |  |  |  |  |
|  |  |  |  |  |  |  |  |  |  |  |  |  |
| Results 2,000ms.  *Note that the number of participants retained in the 2,000ms is low and results have to be interpreted with caution.* | | | | | | | | | | | |  |
|  | | | | | | | | | | | |  |
| Table S3.1 | Mean latencies and errorrates for all indirect measures of Aggression and Sexual Entitlement for 2,000 ms. | | | | | | | | | | |  |
| Figure 4 | Mean Dscores for the IRAP, RRT and IAT for Aggression and Sexual Entitlement 2,000ms. | | | | | | | | | | |  |
| Table S3.2 | Multitrait-multimethod matrix direct and indirect measures of Aggression and Sexual entitlement and additional correlations between study variables 2,000ms. | | | | | | | | | | |  |
| Table S3.3 | Regression analyses direct and indirect measures of Aggression and Sexual entitlement on informant reported aggression and sexual aggressive behavior 2,000ms | | | | | | | | | | |  |
|  |  | | | | | | | | | | |  |
|  |  | | | | | | | | | | |  |
|  |  | | | | | | | | | | |  |
|  |  |  |  |  |  |  |  |  |  |  |  |  |
|  |  |  |  |  |  |  |  |  |  |  |  |  |
|  |  |  |  |  |  |  |  |  |  |  |  |  |

| Table S3.1 |  |  |  |  |  |  |  |
| --- | --- | --- | --- | --- | --- | --- | --- |
| *Mean latencies and errorrates for all indirect measures of Aggression and Sexual Entitlement for 2,000 ms.* | | | | | | |  |
|  |  | Aggression (*N* = 72) | | |  | Sexual entitlement (*N* = 39) | |
|  |  | IRAP | RRT | IAT |  | IRAP | RRT |
| Mean latency in ms | *M* (*SD*) | 1420.81 (254.64) | 12.41.68 (244.31) | 909.04 (186.71) |  | 1787.83 (220.44) | 1601.72 (290.57) |
|  | *t* | *t*(71) = 9.234** *d*= 1.09 | |  |  | *t*(38)= 5.598** *d*= 0.90 | |
|  |  |  | *t*(71) = 14.887** *d*= 1.75 | |  |  |  |
|  |  | *t*(71)= 23.755** *d*= 2.80 | | |  |  |  |
|  |  |  |  |  |  |  |  |
| % Error | *M* (*SD*) | 5.50 (2.78) | 7.03 (4.95) | 3.22 (2.76) |  | 4.11 (2.07) | 4.23 (3.50) |
|  | *t* | *t*(71) = -3.096* *d*= 0.36 | |  |  | *t*(38)= -.0235 | |
|  |  |  | *t*(71)= 7.814** *d*= 0.92 | |  |  |  |
|  |  | *t*(90)= 6.583** *d*= 0.78 | | |  |  |  |
| *_Note._* _Mean latencies and error rates were significantly and positively associated within the same trait (mean latency_ *_r_* _= .64 – 78; percentage error_ *_r_* _= .44 - .55) and across methods and traits (mean latency_ *_r_* _= .42 - .75; percentage error_ *_r_* _= .34 - .57). However, error rates of the Aggression and Sexual entitlement IRAP (_*_r_* _= .21) was not significant._ | | | | | | | |


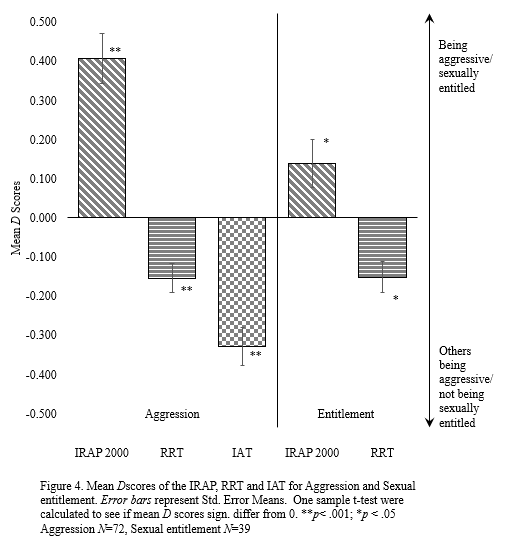

| Table S3.3 |  |  |  |  |  |  |  |  |  |  |  |  |  |  |  |
| --- | --- | --- | --- | --- | --- | --- | --- | --- | --- | --- | --- | --- | --- | --- | --- |
| *Regression analyses direct and indirect measures of Aggression and Sexual entitelement on informant reported aggression and sexual aggressive behavior 2,000ms* | | | | | | | | | | | | | | |  |
| Informant reported aggression | | | | | |  |  | Sexual aggressive behavior | | | | | | |  |
|  |  |  |  |  |  |  |  |  |  |  |  |  |  |  |  |
|  | B (SE) | | 95% CI | | |  |  | B (SE) | | Wald | Exp (B) | 95% CI for Exp(B) | | |  |
| Model 1 |  |  |  |  |  |  | Model 1 |  |  |  |  |  |  |  |  |
| R^2^ | **.36** |  |  |  |  |  | Nagelkerke R^2^ |  |  |  |  |  |  |  |  |
| Constant | **0.91** | **(.19)** | **0.50** | **,** | **1.28** |  | Constant |  |  |  |  |  |  |  |  |
| Aggression | **0.52** | **(.09)** | **0.35** | **,** | **0.71** |  | Sexual entitlement |  |  |  |  |  |  |  |  |
|  |  |  |  |  |  |  |  |  |  |  |  |  |  |  |  |
| Model 2 |  |  |  |  |  |  | Model 2 |  |  |  |  |  |  |  |  |
| ΔR^2^ | .02 |  |  |  |  |  | Nagelkerke R^2^ |  |  |  |  |  |  |  |  |
| Constant | **1.04** | **(.23)** | **0.58** | **,** | **1.53** |  | Constant |  |  |  |  |  |  |  |  |
| Aggression | **0.50** | **(.10)** | **0.31** | **,** | **0.70** |  | Sexual entitlement |  |  |  |  |  |  |  |  |
| *D*score IRAP | -0.02 | (.08) | -0.19 | **,** | 0.13 |  | *D*score IRAP |  |  |  |  |  |  |  |  |
| *D*score RRT | 0.09 | (.15) | -0.20 | , | 0.39 |  | *D*score RRT |  |  |  |  |  |  |  |  |
| *D*score IAT | 0.17 | (.13) | -0.08 | , | 0.44 |  |  |  |  |  |  |  |  |  |  |
| *_Note_*_. Sign. values are in_ **_Bold_**_. Aggression_ *_N_* _= 69; Sexual entitlement_ *_N_* _= 39. Results for aggression were bootstrapped due to the non-normal distribution of Aggression. As both regressionanalyses indicated the presence of multivariate outliers, sensitivity analyses where performed. For the lineair regression analyses results did not differentiate and outliers were retained in the model. For the logistic regression analyses, 2 multivariate outliers were deleted as resulst were influenced. Therefore,_ *_N_* _= 37. However, due to not enough power, errors were detected in the analyses and we were not able to run the analyses._ | | | | | | | | | | | | | | |  |
|  |  |  |  |  |  |  |  |  |  |  |  |  |  |  |  |
|  |  |  |  |  |  |  |  |  |  |  |  |  |  |  |  |
|  |  |  |  |  |  |  |  |  |  |  |  |  |  |  |  |
|  |  |  |  |  |  |  |  |  |  |  |  |  |  |  |  |

| Online supplementary material S4 | | | | | | | | | | | |  |
| --- | --- | --- | --- | --- | --- | --- | --- | --- | --- | --- | --- | --- |
|  |  |  |  |  |  |  |  |  |  |  |  |  |
|  |  |  |  |  |  |  |  |  |  |  |  |  |
|  |  |  |  |  |  |  |  |  |  |  |  |  |
|  |  |  |  |  |  |  |  |  |  |  |  |  |
| Results 5,000ms. | | | | | | | | | | | |  |
|  | | | | | | | | | | | |  |
| Table S4.1 | Mean latencies and errorrates for all indirect measures of Aggression and Sexual Entitlement for 5,000 ms. | | | | | | | | | | |  |
| Figure 4 | Mean Dscores for the IRAP, RRT and IAT for Aggression and Sexual Entitlement 5,000ms. | | | | | | | | | | |  |
| Table S4.2 | Multitrait-multimethod matrix direct and indirect measures of Aggression and Sexual entitlement and additional correlations between study variables 5,000ms. | | | | | | | | | | |  |
| Table S4.3 | Regression analyses direct and indirect measures of Aggression and Sexual entitlement on informant reported aggression and sexual aggressive behavior 5,000ms | | | | | | | | | | |  |
|  |  | | | | | | | | | | |  |
|  |  | | | | | | | | | | |  |
|  |  | | | | | | | | | | |  |
|  |  |  |  |  |  |  |  |  |  |  |  |  |
|  |  |  |  |  |  |  |  |  |  |  |  |  |
|  |  |  |  |  |  |  |  |  |  |  |  |  |

| Table S4.1 |  |  |  |  |  |  |  |
| --- | --- | --- | --- | --- | --- | --- | --- |
| *Mean latencies and errorrates for all indirect measures of Aggression and Sexual Entitlement for 5,000 ms.* | | | | | | |  |
|  |  | Aggression (*N* = 95) | | |  | Sexual entitlement (*N* = 90) | |
|  |  | IRAP | RRT | IAT |  | IRAP | RRT |
| Mean latency in ms | *M* (*SD*) | 1574.63 (404.62) | 1371.22 (373.87) | 967.48 (224.75) |  | 2234.16 (525.05) | 2031.51 (547.93) |
|  | *t* | t(94) = 9.118** *d*= 0.94 | |  |  | t(89)= 6.513** *d*= 0.69 | |
|  |  |  | t(90) = 14.455** *d*= 1.48 | |  |  |  |
|  |  | t(94)= 21.914** *d*= 2.25 | | |  |  |  |
|  |  |  |  |  |  |  |  |
| % Error | *M* (*SD*) | 5.56 (2.94) | 7.42 (5.37) | 2.94 (2.58) |  | 3.94 (2.50) | 3.69 (3.32) |
|  | *t* | t(94) = -4.062** *d*= 0.42 | |  |  | t(89)= 0.783 | |
|  |  |  | t(94)= 9.007** *d*= 0.92 | |  |  |  |
|  |  | t(94)= 8.158** *d*= 0.84 | | |  |  |  |
| *_Note._* _Mean latencies and error rates were significantly and positively associated within the same trait (mean latency_ *_r_*_= .36 – 56; percentage error_ *_r_*_= .69 - .85) and across methods and traits (mean latency_ *_r_*_= .71 - .82; percentage error_ *_r_*_= .21 - .55)._ | | | | | | | |


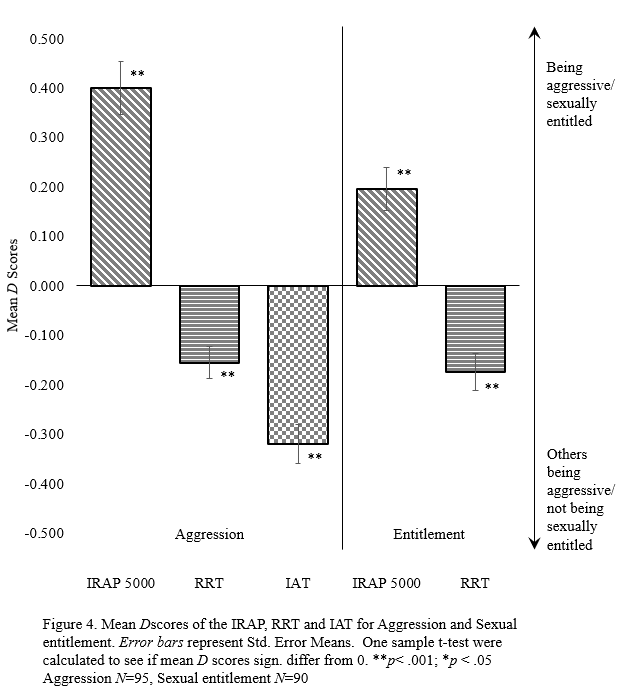

| Online supplementary material S5 | | | | | | | | | | | |  |
| --- | --- | --- | --- | --- | --- | --- | --- | --- | --- | --- | --- | --- |
|  |  |  |  |  |  |  |  |  |  |  |  |  |
|  |  |  |  |  |  |  |  |  |  |  |  |  |
|  |  |  |  |  |  |  |  |  |  |  |  |  |
|  |  |  |  |  |  |  |  |  |  |  |  |  |
| Results for the individual trailtypes of the IRAP *Note that the number of participants retained in the 2,000ms is low and results have to be interpreted with caution.* | | | | | | | | | | | |  |
|  | | | | | | | | | | | |  |
| Figure 5a | Mean Dscores of the individual trial type scores of the IRAP for Aggression 2,000ms | | | | | | | | | | |  |
| Figure 5b | Mean Dscores of the individual trial type scores of the IRAP for Entitlement 2,000ms | | | | | | | | | | |  |
| Table S5.1 | Multitrait-multimethod matrix direct and indirect measures of Aggression and Sexual entitlement and additional correlations between study variables 2,000ms for the individual trailtypes of the IRAP | | | | | | | | | | |  |
| Table S5.2 | Regression analyses direct and indirect measures of Aggression and Sexual entitlement on informant reported aggression and sexual aggressive behavior 2,000ms individual trailtypes | | | | | | | | | | |  |
|  |  |  |  |  |  |  |  |  |  |  |  |  |
| Figure 5a | Mean Dscores of the individual trial type scores of the IRAP for Aggression 3,000ms | | | | | | | | | | |  |
| Figure 5b | Mean Dscores of the individual trial type scores of the IRAP for Entitlement 3,000ms | | | | | | | | | | |  |
| Table S5.3 | Multitrait-multimethod matrix direct and indirect measures of Aggression and Sexual entitlement and additional correlations between study variables 3,000ms for the individual trailtypes of the IRAP | | | | | | | | | | |  |
| Table S5.4 | Regression analyses direct and indirect measures of Aggression and Sexual entitlement on informant reported aggression and sexual aggressive behavior 3,000ms individual trailtypes | | | | | | | | | | |  |
|  |  |  |  |  |  |  |  |  |  |  |  |  |
| Figure 5a | Mean Dscores of the individual trial type scores of the IRAP for Aggression 5,000ms | | | | | | | | | | |  |
| Figure 5b | Mean Dscores of the individual trial type scores of the IRAP for Entitlement 5,000ms | | | | | | | | | | |  |
| Table S5.5 | Multitrait-multimethod matrix direct and indirect measures of Aggression and Sexual entitlement and additional correlations between study variables 5,000ms for the individual trailtypes of the IRAP | | | | | | | | | | |  |
| Table S5.6 | Regression analyses direct and indirect measures of Aggression and Sexual entitlement on informant reported aggression and sexual aggressive behavior 5,000ms individual trailtypes | | | | | | | | | | |  |
|  |  |  |  |  |  |  |  |  |  |  |  |  |

| Online supplementary material S5 |  |
| --- | --- |
|  |  |
|  |  |
|  |  |
|  |  |
| Results for the individual trailtypes of the IRAP |  |
| Different Trial types of the IRAP (3,000 ms)  To give a full overview, we also looked at the descriptive statistics and validity of the different IRAP trial types, namely I am aggressive, Others are aggressive, I am peaceful, Others are peaceful, and I am sexually entitled, I am not sexually entitled, Women have to obey to a man’s sexual needs and Women can make their own decisions. When looking at the different trial types of the IRAP, mean scores showed a positive bias for I am aggressive, and Others are aggressive (D = 0.89, 0.79, respectively) and a negative bias for I am peaceful (D = -0.17). The average D-score for Others are peaceful was not significantly different from 0. For sexual entitlement, mean scores were positive for I am sexually entitled, Women can make their own decisions and Women have to obey to a man’s sexual needs (D = 0.33, 0.20, and 0.17, respectively). No effect for I am not sexually entitled was found.  Validity. With regard to convergent validity, the positive monotrait-heteromethod association between the RRT and the IRAP was mostly based on the trial type I am aggressive, I am sexually entitled and Women have to obey a man’s sexual needs. In contrast, the association with self-reported aggression was mostly based on Others are aggressive, I am peaceful, and I am sexually entitled for aggression and sexual entitlement, respectively. For the aggression trial types, correlations with other indirect measures were never (significantly) higher than the correlations with the direct measures. For entitlement, the correlations with the RRT were (slightly) higher, although, not significantly higher than the correlations with the direct measure (ps > .05).  Concerning discriminant validity, the same patterns as for the compound D-score was found. In general, monotrait-heteromethod correlations were higher than heterotrait-heteromethod correlations (the only exception was found for Others are peaceful). Only for I am sexually entitled, associations with the Sexual Entitlement RRT (z = 1.85, p= .03) and self-reported sexual entitlement (z = 2.61, p< .05) were significantly higher. In line with the results of the compound D-score, there was a possible method factor for the IRAP trial types as only for I am sexually entitled and most of I am aggressive, monotrait-heteromethod associations were consistently and significantly higher than the heterotrait-monotrait associations. Except for the Sexual Entitlement RRT and Others are peaceful associations, the heterotrait-heteromethod correlations were low as expected. Concerning the other associations, opposite to the compound D-score, there was a significant positive association between Others are aggressive, Women have to obey a man’s sexual needs, and age.  In line with the aggression IRAP compound D-score, the individual trial types did not significantly explain informant reported aggression. For sexual entitlement, however, endorsement of the trial type Women have to obey a man’s sexual needs had a significantly positive association with sexual aggressive behavior (Odds ratio = 3.15). |  |
|  |  |
|  |  |
|  |  |
|  |  |
|  |  |
|  |  |
|  |  |
|  |  |
|  |  |
|  |  |
|  |  |
|  |  |
|  |  |
|  |  |
|  |  |
|  |  |
|  |  |
|  |  |
|  |  |
|  |  |
|  |  |
|  |  |
|  |  |
|  |  |
|  |  |
|  |  |
|  |  |
|  |  |
|  |  |
|  |  |


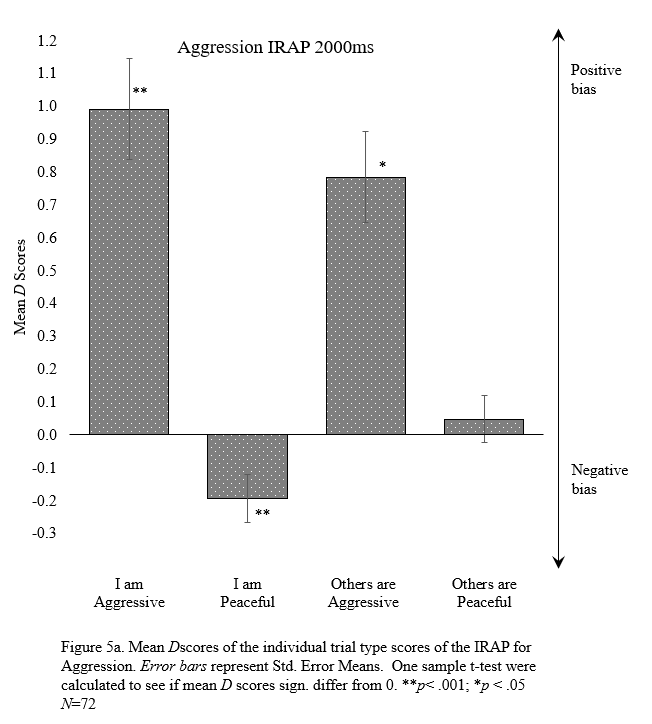


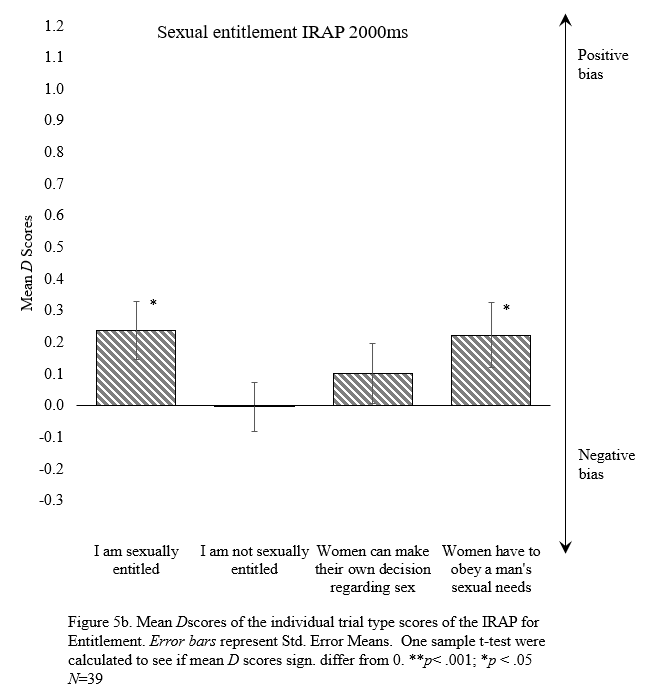

| Table S5.1 (Continued). |  |  |  |  |  |  |  |  |  |  |  |
| --- | --- | --- | --- | --- | --- | --- | --- | --- | --- | --- | --- |
|  | | | | | | | | | | |  |
|  |  | Indirect measures | | | | | | | | |  |
|  |  | IRAP Aggression | | | |  | IRAP Sexual Entitlement | | | |  |
|  |  | 1^a^ | 2^a^ | 3 | 4 |  | 1 | 2^a^ | 3 | 4 |  |
| Informant reported aggression | *r* | .09 | .08 | .29* | -.09 |  | -.16 | -.20 | -.12 | -.19 |  |
|  | *n* | 69 | 69 | 69 | 69 |  | 38 | 38 | 38 | 38 |  |
| Sexual aggressive behavior | *r* | .06 | .08 | -.04 | .01 |  | .15 | .07 | -.12 | .09 |  |
|  | *n* | 72 | 72 | 72 | 72 |  | 39 | 39 | 39 | 39 |  |
| IM | *r* | -.08 | -.20 | -.23* | .04 |  | -.08 | -.30 | .09 | .15 |  |
|  | *n* | 72 | 72 | 72 | 72 |  | 39 | 39 | 39 | 39 |  |
| SDE | *r* | .00 | -.01 | -.22 | -.07 |  | .17 | -.21 | .22 | .15 |  |
|  | *n* | 72 | 72 | 72 | 72 |  | 39 | 39 | 39 | 39 |  |
| Age | *r* | -.13 | .18 | -.02 | -.21 |  | .12 | .08 | -.04 | .39* |  |
|  | *n* | 71 | 71 | 71 | 71 |  | 39 | 39 | 39 | 39 |  |
| *_Note_*_. Sexual aggressive behavior is a dummy variable with having shown sexually agressive behavior serving as the reference group. aDue to non-normal distribution Spearman Rho was calculated. **Correlation is significant at the .001 level (2-tailed). *Correlation is significant at the .05 level (2-tailed). For a more clear visual overview of the MTMM analyses, the variable Sexual Entitlement IAT was included in the table._ | | | | | | | | | | |  |
|  |  |  |  |  |  |  |  |  |  |  |  |
|  |  |  |  |  |  |  |  |  |  |  |  |
|  |  |  |  |  |  |  |  |  |  |  |  |
|  |  |  |  |  |  |  |  |  |  |  |  |
|  |  |  |  |  |  |  |  |  |  |  |  |
|  |  |  |  |  |  |  |  |  |  |  |  |
|  |  |  |  |  |  |  |  |  |  |  |  |
|  |  |  |  |  |  |  |  |  |  |  |  |
|  |  |  |  |  |  |  |  |  |  |  |  |

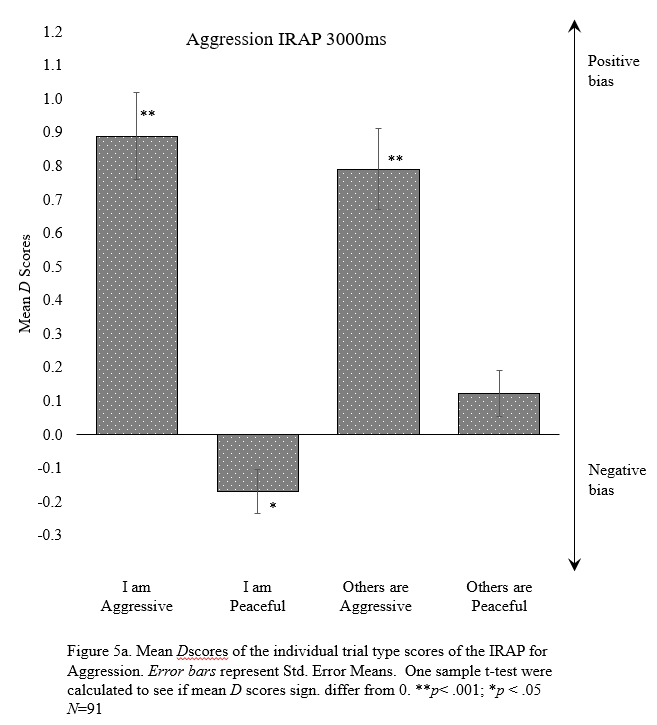


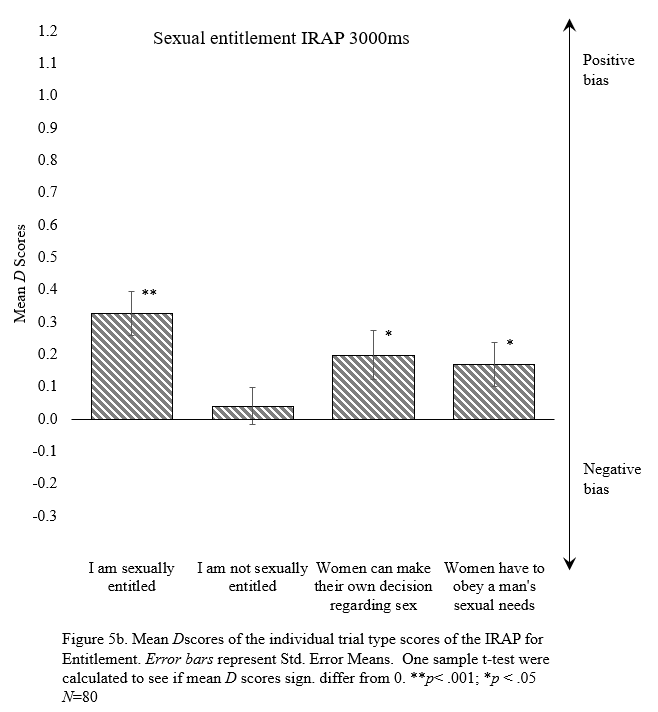

| Table S5.3 (Continued). |  |  |  |  |  |  |  |  |  |  |  |
| --- | --- | --- | --- | --- | --- | --- | --- | --- | --- | --- | --- |
|  | | | | | | | | | | |  |
|  |  | Indirect measures | | | | | | | | |  |
|  |  | IRAP Aggression | | | |  | IRAP Sexual Entitlement | | | |  |
|  |  | 1^a^ | 2^a^ | 3 | 4 |  | 1 | 2 | 3 | 4 |  |
| Informant reported aggression | *r* | .04 | -.02 | .14 | -.08 |  | .00 | -.08 | -.12 | -.10 |  |
|  | *n* | 84 | 84 | 84 | 84 |  | 77 | 77 | 77 | 77 |  |
| Sexual aggressive behavior | *r* | -.05 | .17 | -.06 | .08 |  | .21 | .01 | -.03 | .21 |  |
|  | *n* | 91 | 91 | 91 | 91 |  | 80 | 80 | 80 | 80 |  |
| IM | *r* | -.07 | -.09 | -.12 | .07 |  | -.10 | -.11 | .10 | .06 |  |
|  | *n* | 91 | 91 | 91 | 91 |  | 80 | 80 | 80 | 80 |  |
| SDE | *r* | .00 | -.01 | -.14 | -.11 |  | .05 | -.07 | .15 | .01 |  |
|  | *n* | 91 | 91 | 91 | 91 |  | 80 | 80 | 80 | 80 |  |
| Age | *r* | -.19 | .28* | .00 | -.01 |  | .06 | .06 | .00 | .31* |  |
|  | *n* | 90 | 90 | 90 | 90 |  | 79 | 79 | 79 | 79 |  |
| *_Note_*_. Sexual aggressive behavior is a dummy variable with having shown sexually agressive behavior serving as the reference group. aDue to non-normal distribution Spearman Rho was calculated. **Correlation is significant at the .001 level (2-tailed). *Correlation is significant at the .05 level (2-tailed). For a more clear visual overview of the MTMM analyses, the variable Sexual Entitlement IAT was included in the table._ | | | | | | | | | | |  |
|  |  |  |  |  |  |  |  |  |  |  |  |
|  |  |  |  |  |  |  |  |  |  |  |  |
|  |  |  |  |  |  |  |  |  |  |  |  |
|  |  |  |  |  |  |  |  |  |  |  |  |
|  |  |  |  |  |  |  |  |  |  |  |  |
|  |  |  |  |  |  |  |  |  |  |  |  |
|  |  |  |  |  |  |  |  |  |  |  |  |
|  |  |  |  |  |  |  |  |  |  |  |  |
|  |  |  |  |  |  |  |  |  |  |  |  |

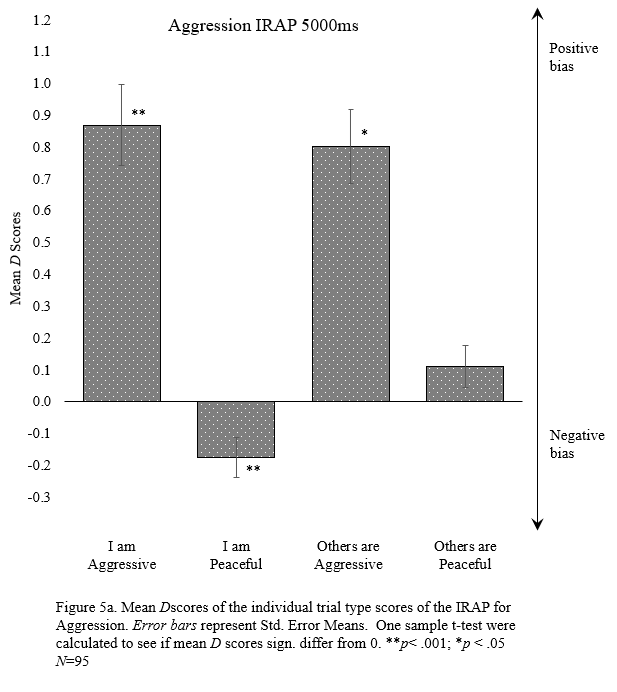


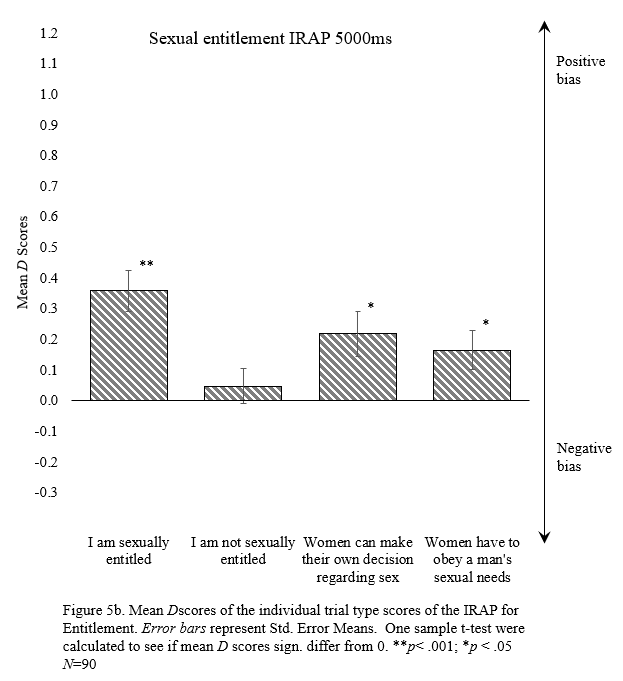

| Table S5.5 (Continued). |  |  |  |  |  |  |  |  |  |  |  |
| --- | --- | --- | --- | --- | --- | --- | --- | --- | --- | --- | --- |
|  | | | | | | | | | | |  |
|  |  | Indirect measures | | | | | | | | |  |
|  |  | IRAP Aggression | | | |  | IRAP Sexual Entitlement | | | |  |
|  |  | 1^a^ | 2^a^ | 3 | 4 |  | 1 | 2 | 3 | 4 |  |
| Informant reported aggression | *r* | .11 | .05 | .15 | -.07 |  | -.03 | -.10 | -.09 | -.08 |  |
|  | *n* | 88 | 88 | 88 | 88 |  | 85 | 85 | 85 | 85 |  |
| Sexual aggressive behavior | *r* | .02 | .09 | -.03 | .08 |  | .20 | -.01 | -.02 | .18 |  |
|  | *n* | 95 | 95 | 95 | 95 |  | 90 | 90 | 90 | 90 |  |
| IM | *r* | -.09 | -.18 | -.12 | .04 |  | -.07 | -.09 | -.01 | .00 |  |
|  | *n* | 95 | 95 | 95 | 95 |  | 90 | 90 | 90 | 90 |  |
| SDE | *r* | .01 | -.02 | -.15 | -.12 |  | .04 | -.08 | .06 | -.02 |  |
|  | *n* | 95 | 95 | 95 | 95 |  | 90 | 90 | 90 | 90 |  |
| Age | *r* | -.14 | .26* | .03 | -.02 |  | .07 | .10 | -.06 | .30** |  |
|  | *n* | 94 | 94 | 94 | 94 |  | 89 | 89 | 89 | 89 |  |
| *_Note_*_. Sexual aggressive behavior is a dummy variable with having shown sexually agressive behavior serving as the reference group. aDue to non-normal distribution Spearman Rho was calculated. **Correlation is significant at the .001 level (2-tailed). *Correlation is significant at the .05 level (2-tailed). For a more clear visual overview of the MTMM analyses, the variable Sexual Entitlement IAT was included in the table._ | | | | | | | | | | |  |
|  |  |  |  |  |  |  |  |  |  |  |  |
|  |  |  |  |  |  |  |  |  |  |  |  |
|  |  |  |  |  |  |  |  |  |  |  |  |
|  |  |  |  |  |  |  |  |  |  |  |  |
|  |  |  |  |  |  |  |  |  |  |  |  |
